# The perinecrotic niche of glioblastoma drives tumor-associated macrophage polarization and immunosuppression via podoplanin-mediated CLEC5A activation

**DOI:** 10.1172/JCI199228

**Published:** 2026-06-02

**Authors:** Jiabo Li, Xuya Wang, Luqing Tong, Bo Feng, Ling-kai Shih, Steven M. Markwell, Hannah Nuszen, Tomasz Gruchala, Nicholas G. Lam, Petros Basakis, Erika Ruiz-Yamamoto, Deyu Fang, Roger Stupp, Xuejun Yang, Daniel J. Brat

**Affiliations:** 1 Department of Pathology, Northwestern Medicine Malnati Brain Tumor Institute of the Robert H. Lurie Comprehensive Cancer Center, Northwestern University Feinberg School of Medicine, Chicago, Illinois, USA.; 2 Department of Neurosurgery, Tsinghua University Beijing Tsinghua Changgung Hospital, Beijing, China.; 3 Department of Neurosurgery, The First Affiliated Hospital, Zhejiang University School of Medicine, Hangzhou, Zhejiang, China.; 4 Department of Dermatology, The Fourth Affiliated Hospital, Zhejiang University School of Medicine, Yiwu, Zhejiang, China.; 5 Department of Pathology, Center for Human Immunobiology and Robert H. Lurie Comprehensive Cancer Center, and; 6 Departments of Neurological Surgery and Neurology, Northwestern Medicine Malnati Brain Tumor Institute of the Robert H. Lurie Comprehensive Cancer Center, Northwestern University Feinberg School of Medicine, Chicago, Illinois, USA.

**Keywords:** Neuroscience, Oncology, Brain cancer, Hypoxia, Macrophages

## Abstract

Glioblastoma (GBM), isocitrate dehydrogenase-WT (IDH-WT) (WHO grade 4) is the most common malignant glioma in adults and is characterized by a hypoxic and immunosuppressive tumor microenvironment (TME). Bone marrow–derived tumor-associated macrophages (TAMs) dominate the immune landscape in GBM and are recruited to the perinecrotic niche following the onset of necrosis. C-type lectin domain–containing 5A (*CLEC5A*) has the strongest association with poor clinical outcomes among immune-related genes in GBM and is preferentially expressed in hypoxic, perinecrotic TAMs. *CLEC5A* overexpression promotes TAM polarization toward an immunosuppressive phenotype and secretion of immunoregulatory cytokines. Using the replication-competent avian sarcoma-leukosis virus long terminal repeat with a splice acceptor (RCAS)/tumor virus A (tv-a) system GBM model with bone marrow transplantation from *Clec5a^–/–^* donor mice, we demonstrated that CLEC5A loss prolonged survival, delayed tumor progression, and attenuated TME immunosuppression. Mechanistically, podoplanin (PDPN) expressed on glioma cells directly engaged CLEC5A and triggered downstream Syk/JAK/STAT3 signaling in TAMs. Pharmacologic Syk inhibition suppressed glioma growth, diminished TAM infiltration and polarization, reversed the immunosuppressive TME, and prolonged survival in vivo. Collectively, our findings indicate that the PDPN/CLEC5A/Syk/STAT3 axis orchestrates TAM polarization and TME immunosuppression in the perinecrotic niche of GBM, highlighting CLEC5A/Syk as a promising therapeutic target for reversing the immunosuppressive TME and improving outcomes.

## Abstract 2

Glioblastoma cells suppress anti-tumor immunity by activating macrophages through the PDPN-CLEC5A-Syk pathway, revealing a potential therapeutic target for reversing immunosuppression.

## Graphical abstract

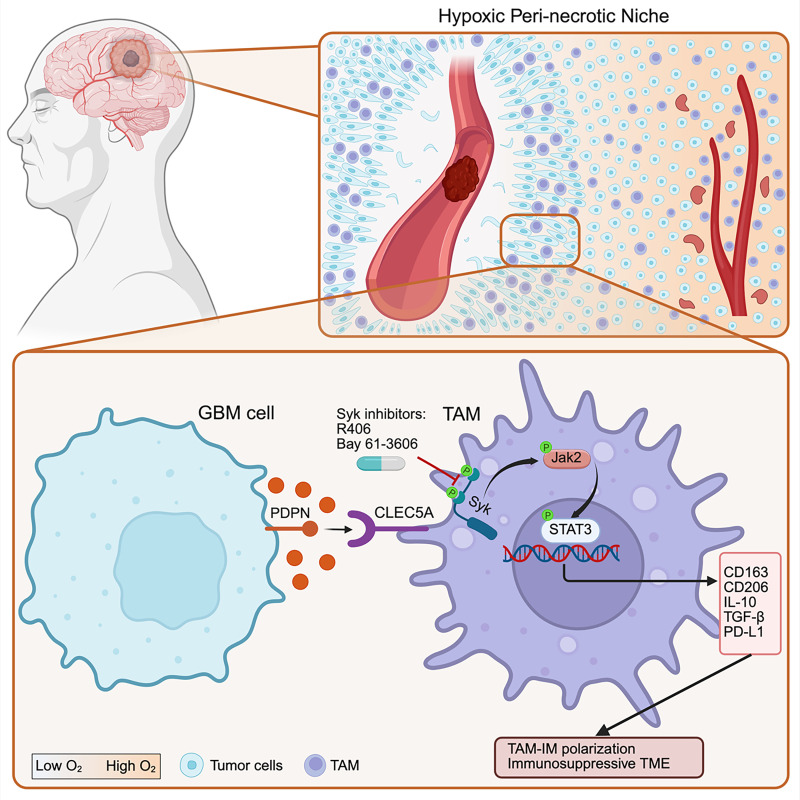

## Introduction

Glioblastoma (GBM), isocitrate dehydrogenase-WT (IDH-WT) (WHO grade 4) is the most frequent and lethal malignant brain tumor of adults (1). Despite standard treatment that includes maximal safe resection, radiotherapy, and temozolomide (TMZ) chemotherapy, the median overall survival is only 15 months, with a 5-year survival rate below 7% (1, 2). Obstacles to developing more effective therapies include spatial heterogeneity and subclonal evolution, the blood-brain barrier (BBB), glioma stem cell (GSC) therapeutic resistance, and, critically, an immunosuppressive tumor microenvironment (TME).

The GBM TME is highly dynamic and undergoes extensive restructuring during tumor progression. Multiple factors contribute to immune remodeling and shape immune cell recruitment, activation states, and functional polarization within the TME, including tumor growth, metabolic stress, angiogenic and stromal remodeling, oncogenic context–dependent cytokine and chemokine programs, therapy-induced changes, and hypoxia and necrosis, particularly within the perinecrotic niche (3–5). Tumor-associated macrophages (TAMs) are the most abundant non-neoplastic cells in the GBM TME, accounting for 30%–50% of total cells, and increase dramatically with the onset of necrosis, with a migration pattern from the vasculature toward the perinecrotic niche (3, 6). Approximately 85% of TAMs are bone marrow–derived monocytes (BMDMs) that differentiate into macrophages upon extravasation into the glioma parenchyma, while the remaining 15% are resident microglia (7). TAMs exhibit a continuum of phenotypes, ranging from inflammatory and tumor-suppressive (TAM-INF; M1-like) to immunosuppressive and tumor-promoting (TAM-IM; M2-like) and have recently been divided into 7 transcriptional subsets with variable immunosuppressive qualities (6, 8, 9). TAM-IMs strongly contribute to immune escape and glioma progression, at least in part through secretion of cytokines that include IL-10, pleiotrophin (PTN), CCL17, CCL22, and TGF-β, yet there is a need to uncover molecular regulators that drive TAM polarization and the immunosuppressive response (10, 11).

C-type lectin domain–containing 5A (CLEC5A), also known as myeloid DAP12–associating lectin 1 (MDL-1), is a type II transmembrane protein belonging to the C-type lectin superfamily and is exclusively expressed by myeloid-derived cells, supporting their activation, differentiation, and survival (12, 13). CLEC5A has been most thoroughly studied as a regulator of inflammatory responses to viral infectious diseases, in which it signals through the adaptor DNAX-activating protein 12 (DAP12) to activate downstream pathways (14, 15). In cancer, CLEC5A has been investigated as a prognostic biomarker and appears to regulate cell proliferation and migration through PI3K/AKT/mTOR signaling (16–18). Notably, *CLEC5A* has been identified as a marker of proangiogenic TAMs, a hypoxic and highly immunosuppressive form (9). Upstream signals that may activate CLEC5A in TAMs in the GBM TME have not been defined.

Among candidate ligands capable of engaging C-type lectin receptors, podoplanin (PDPN) — also known as PA2.26, gp38, and gp36 — is a type I integral membrane glycoprotein with diverse distribution in human tissues (19). It interacts with CLEC2 on platelets, mediating activation and aggregation through Src/Syk signaling (20, 21). In glioma, PDPN also has a regulatory role in tumor cell proliferation, epithelial-mesenchymal transition, invasion, and metastasis (22). A subset of GSCs that are radiation resistant express PDPN, and bioinformatics approaches have shown a relationship between PDPN and immune regulation within the TME (19, 21). Collectively, together with the spatial enrichment of both CLEC5A-expressing TAMs and hypoxic GBM cells in perinecrotic regions, these relationships raise the possibility that hypoxia-induced PDPN expression in tumor cells may engage CLEC5A signaling in myeloid cells to cause polarization and immune suppression. Here, we investigate the role of the PDPN/CLEC5A/Syk axis in shaping TAM function within the GBM TME, with a focus on the hypoxic, perinecrotic niches.

## Results

### CLEC5A expression by TAM-IM is a strong predictor of poor clinical outcomes.

**Figure 1:**
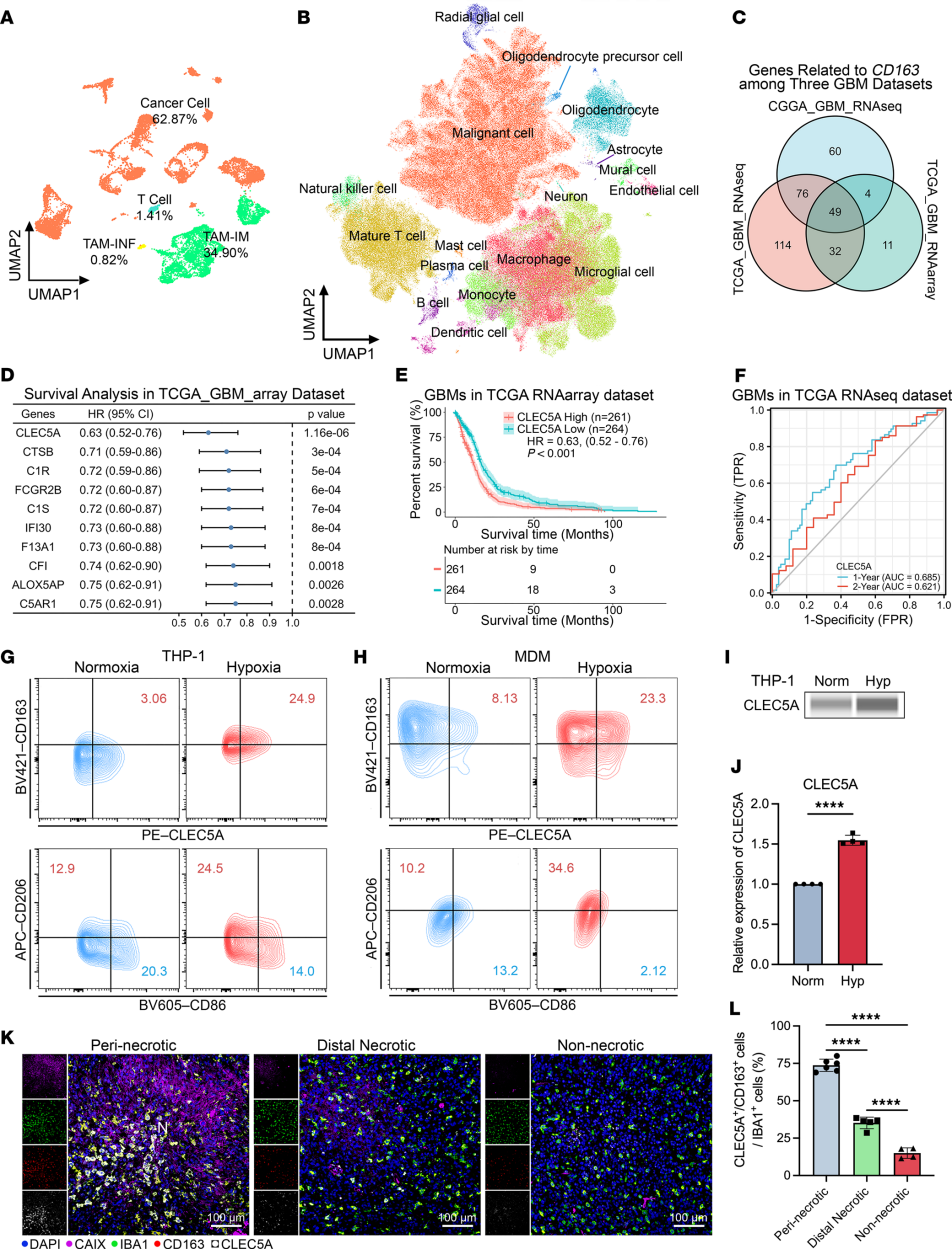
CLEC5A is preferentially expressed by hypoxic TAM-IMs within the perinecrotic niche and correlates with a poor prognosis. (**A**) scRNA-seq Seurat and UMAP analysis clustered samples into 30 distinct gene expression profiles. Four distinct cell subpopulations were identified as follows: cancer cells (*GAP43^+^*, *GPM6B^+^*, *PTN^+^*, *GFAP^+^*, *SOX2^+^*, *OLIG2^+^*, *CD44^+^*, *CCND2^+^*, and *PARP1^+^*); TAM-IMs (*AIF^+^*, *CD68^+^*, *CD163^+^*, and *MSR1^+^*); TAM-INFs (*AIF^+^*, *CD68^+^*, *CD86^+^*, and *CD163^–^*); and T cells (*CD3G^+^*). (**B**) UMAP visualization of a large-scale human GBM scRNA-seq dataset (>338,000 cells), showing major cell populations including neoplastic cells, myeloid cells, lymphoid cells, and vascular and glial cell lineages. (**C**) Spearman correlation reveals 49 genes highly associated with *CD163* among 3 independent GBM datasets. (**D**) Forest plot shows the top CD163-related genes, together with their association with survival. *CLEC5A* had the highest association with survival in TCGA RNA microarray dataset. (**E**) Kaplan-Meier overall survival for patients with GBM with high versus low *CLEC5A* expression in TCGA RNA microarray dataset. (**F**) Time-dependent ROC curves display a *CLEC5A* expression predictive capacity for 1- and 2-year survival of patients with GBM in TCGA database. (**G**) Representative flow cytometric plots of CLEC5A^+^CD163^+^, CD206^+^CD86^–^, and CD206^–^CD86^+^ THP-1 cell populations under normoxia versus hypoxia. (**H**) Representative flow cytometric plots and quantification of CLEC5A^+^CD163^+^, CD206^+^CD86^–^, and CD206^–^CD86^+^ MDM populations under normoxia versus hypoxia. (**I** and **J**) THP-1 protein expression levels of CLEC5A in normoxic (Norm) and hypoxic (Hyp) conditions detected by Simple Western (Jess) (unpaired, 2-tailed Student’s *t* test). (**K**) IF staining for CAIX, IBA1, CLEC5A, and CD163 performed on perinecrotic and distal necrotic regions in necrotic GBM, as well as non-necrotic regions in non-necrotic tumor. Scale bars: 100 μm. Original magnification, ×20. (**L**) Histogram depicting the proportion of CLEC5A^+^CD163^+^ cells relative to IBA1^+^ cells across 3 distinct areas: perinecrotic areas, distal necrotic zones in necrotic tumors, and in non-necrotic gliomas, by IF staining (1-way ANOVA with Tukey’s multiple-comparison test). ****P < 0.0001. All quantitative data are presented as mean ± SD.

To characterize TME profiles of IDH-WT GBM at the single-cell level, we obtained single-cell RNA-seq (scRNA-seq) data from the Gene Expression Omnibus (GEO), which collected 16,201 single cells from 9 IDH-WT GBMs (23). Following established approaches, we first separated neoplastic from non-neoplastic compartments and then annotated major lineages, focusing on myeloid cell populations relevant to our hypothesis (Supplemental Figure 1, A–C; supplemental material available online with this article; https://doi.org/10.1172/JCI199228DS1) (23, 24). Within the myeloid compartment, TAM programs consistent with an immunosuppressive phenotype TAM-IM accounted for approximately 35% of all cells, whereas TAM-INF made up 1% (Figure 1A and Supplemental Figure 1, A–E).

To strengthen cell-type resolution beyond this initial dataset, we further analyzed a larger, independent GBM scRNA-seq atlas (GBmap) (25), which contains more than 338,000 cells and provides a harmonized hierarchical annotation scheme from broad compartments (level 1: neoplastic vs. non-neoplastic) to progressively refined lineages and states (levels 2–4) (Figure 1B and Supplemental Figure 1F). This atlas confirmed that myeloid cells are a major component of the GBM TME, comprising approximately 40% of all cells, and enabled higher-resolution separation of immune lineages and myeloid states, supporting the downstream gene expression analyses.

We next sought to uncover potential biological mediators associated with the TAM-IM phenotype and adverse clinical outcome. Using TCGA and the Chinese Glioma Genome Atlas (CGGA) transcriptional cohorts, we first defined a *CD163*^hi^ macrophage–associated gene module and identified 49 genes consistently coexpressed with *CD163* across datasets (Figure 1C, Supplemental Figure 2, A and B, and Supplemental Table 1). In survival analysis using clinical outcomes from the largest cohort (TCGA GBM array data), *CLEC5A* emerged as the gene most strongly associated with short overall survival among this TAM-related module (Figure 1D and Supplemental Table 1). scRNA-seq data showed that *CLEC5A* was primarily expressed on TAM-IM, similar to *CD163* (Supplemental Figure 2, C and D), with concordant patterns observed in the dependent GBmap atlas (Supplemental Figure 2E). We then verified the relationship between *CLEC5A* and overall survival in patients with GBM using Kaplan-Meier analyses stratifying tumors by median *CLEC5A* expression, which showed that shorter survival times were associated with high *CLEC5A* expression (Figure 1E, Supplemental Figure 2F, and Supplemental Table 2). The results were similar when all patients with diffuse glioma were considered (Supplemental Figure 2G). Although *CD163* was also associated with shorter overall survival, *CLEC5A* demonstrated stronger performance in the same cohorts (Supplemental Figure 2I). Time-dependent receiver operating characteristic (ROC) curves further supported the prognostic value of *CLEC5A* for 1-year (AUC = 0.685) and 2-year (AUC = 0.621) survival status for patients with GBM (Figure 1F) and demonstrated the highest predictive accuracy of all 49 TAM-related genes (Supplemental Table 1). *CLEC5A* expression also predicted the 1-year (AUC = 0.866 in TCGA, AUC = 0.705 in CGGA), 3-year (AUC = 0.841 in TCGA, AUC = 0.728 in CGGA), and 5-year (AUC = 0.795 in TCGA, AUC = 0.734 in CGGA) survival status for all patients with glioma (Supplemental Figure 2H). Collectively, these analyses indicate that CLEC5A expression by TAM-IM is a strong predictor of poor clinical outcomes in patients with GBM.

### CLEC5A is preferentially expressed by hypoxic TAM-IM within the perinecrotic niche.

Given the enrichment of CLEC5A in TAM-IMs and its association with adverse outcomes, we next investigated whether CLEC5A expression is linked to hypoxia and the perinecrotic niche. We analyzed scRNA-seq data using the MsigDB Hallmark Hypoxia (Hypoxia_HM) gene signature to quantify hypoxia programs at the single-cell level (Supplemental Figure 3A). Among TAMs, those cells with higher *CLEC5A* expression had substantially higher Hallmark Hypoxia scores (Supplemental Figure 3B). Conversely, when we divided TAM-IM into *CLEC5A*^+^ and *CLEC5A*^–^ groups on the basis of scRNA-seq data, GSEA further confirmed that CLEC5A-positive TAM-IM were enriched for the “Hypoxia” gene signature (normalized enrichment score [NES] = 1.262, adjusted *P* value [*P*.adj] = 0.003, Supplemental Figure 3C). Consistently, in the GBmap scRNA-seq data, CLEC5A was preferentially enriched in Mono hypoxia and TAM-BDM hypoxia/MES states (Supplemental Figure 2E). To assess spatial regional variations in *CLEC5A* expression, we analyzed Ivy Glioblastoma Atlas Project (GAP) data, which includes transcriptional data from 270 samples within 7 anatomical subregions. We found that *CLEC5A*, TAM-IM markers (*CD163*, *MSR1*, *IL6*, *IL10*), and mesenchymal markers (*CD44*, *CHI3L1*, *CXCR4*, *TIMP1*, *STAT3*, *NFKB1*) were highly expressed in perinecrotic zones and pseudopalisading cells around necrosis, whereas glioma and proneural markers were mainly expressed in infiltrating tumor and cellular tumor structures (Supplemental Figure 3D).

To further determine if *CLEC5A* transcriptional upregulation corresponded to increased protein expression, we exposed phorbol 12-myristate 13-acetate–activated (PMA-activated) human monocytic leukemia cell line THP-1 cells and macrophage-CSF–activated human monocyte-derived macrophages (MDMs) to normoxic (21% O_2_) and hypoxic (1% O_2_) for 48 hours and quantified CLEC5A by flow cytometry and Simple Western (Jess). Hypoxia significantly increased CLEC5A protein abundance, expanded the proportion of CLEC5A^+^CD163^+^ and CD206^+^ macrophages, and decreased CD86^+^ expression relative to normoxia (Figure 1, G–J, and Supplemental Figure 3, E and F), supporting a hypoxia-associated induction of a CLEC5A^+^ TAM-IM phenotype in vitro.

We then examined whether this hypoxia-linked CLEC5A pattern was present in human GBM samples and, in particular, whether it was localized to perinecrotic regions. In multiplex immunofluorescence (IF) staining analyses, we incorporated the hypoxia marker carbonic anhydrase IX (CAIX) and found that CLEC5A and CD163 were coexpressed in a subset of IBA1^+^ myeloid cells and preferentially enriched in the CAIX^+^ perinecrotic niches, with diminishing density in CAIX^–^ regions more distal to necrosis and reduced expression in non-necrotic tumors (Figure 1, K and L, and Supplemental Figure 3, G and H). Finally, to investigate variation of CLEC5A expression across GBM spatial domains, we neurosurgically sampled tissue from 22 patients with GBM and defined anatomic subregions as perinecrotic regions (PRs), contrast-enhancing regions (ERs), and infiltrating regions (IRs) on the basis of MRI (Supplemental Figure 3I). We found that CLEC5A expression was higher in the PR region than in the ER and IR regions (Supplemental Figure 3, J and K), consistent with preferential localization to the perinecrotic niche. Collectively, these results indicate that CLEC5A is expressed by hypoxia-associated TAM-IMs and is enriched in the hypoxic, perinecrotic niches of GBM.

### CLEC5A enhances TAM polarization toward a TAM-IM phenotype.

To experimentally address the biological role of CLEC5A in the GBM TME, we performed a series of in vitro and in vivo experiments to determine its influence on TAM polarization. We stably knocked down (sh-CLEC5A-1 or sh-CLEC5A-2) or overexpressed (OE-CLEC5A) *CLEC5A* in THP-1 cells and verified transfection efficiency using quantitative PCR (qPCR) and IF. *CLEC5A* knockdown led to mRNA expression levels that were approximately 20%–30% of control levels, while overexpression led to a 20-fold increase, which was within the range observed under hypoxic induction (Supplemental Figure 4, A and B). Importantly, a cell counting Kit-8 assay showed no significant differences in proliferation among control, sh-PDPN, and OE-CLEC5A THP-1 cells (Supplemental Figure 4C).

**Figure 2:**
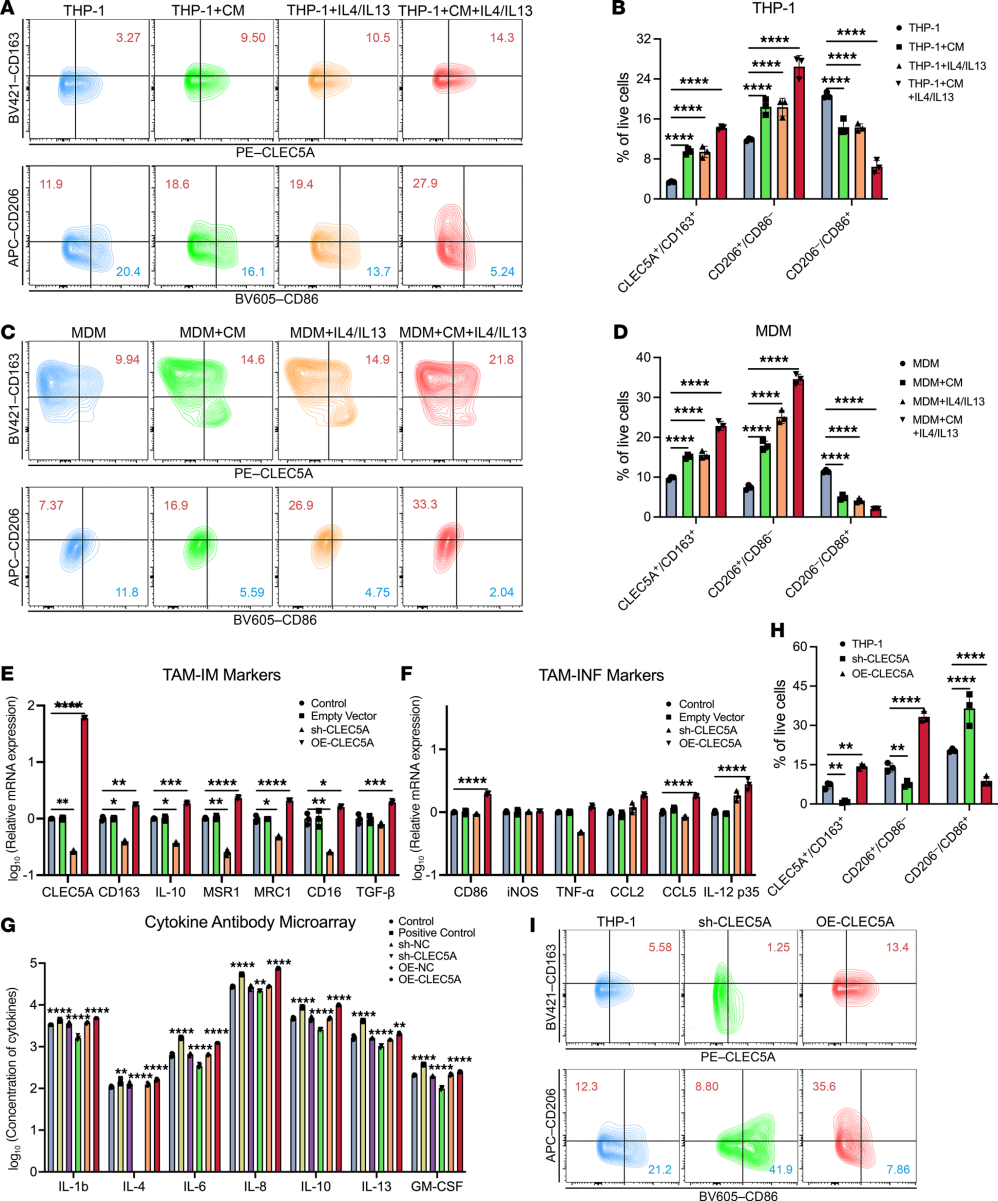
CLEC5A enhances TAM polarization toward a TAM-IM phenotype. (**A** and **B**) Representative flow cytometric plots and quantification of CLEC5A^+^CD163^+^, CD206^+^CD86^–^, and CD206^–^CD86^+^ THP-1 cell populations cocultured with GBM neurosphere CM, IL-4 and IL-13 stimulation, or combined CM plus IL-4 and IL-13 treatment. (**C** and **D**) Representative flow cytometry plots and quantification of CLEC5A^+^CD163^+^, CD206^+^CD86^–^, and CD206^–^CD86^+^ MDM populations cocultured with GBM neurosphere CM, IL-4/IL-13 stimulation, or combined CM plus IL-4/IL-13 treatment. (**E** and **F**) qPCR showing TAM-IM markers (*CLEC5A*, *CD163*, *IL10*, *MSR1*, *MRC1*, *CD16*, and *TGFB*) and TAM-INF markers (*CD86*, *iNOS*, *TNFA*, *CCL2*, *CCL5*, and *IL12p35*) in THP-1 cells after CLEC5A knockdown or overexpression. (**G**) Cytokine antibody microarray showing IL-1β, IL-4, IL-6, IL-8, IL-10, IL-13, and GM-CSF expression in THP-1 cells after CLEC5A knockdown or overexpression. (**H** and **I**) Representative flow cytometry plots and quantification of TAM polarization markers in THP-1 cells following CLEC5A knockdown or overexpression (THP-1 cells were cocultured with NU02068 neurospheres). *P < 0.05, **P < 0.01, ***P < 0.001, and ****P < 0.0001, by 2-way ANOVA followed by Dunnett’s multiple-comparison test (**B** and **D**–**H**).

To examine whether CLEC5A expression tracked with TAM-IM polarization cues commonly present in GBM, IL-4 and IL-13 were used to induce an immunosuppressive phenotype in PMA-activated THP-1 cells and/or cells were treated with glioma conditioned media (CM). Flow cytometry and qPCR assays showed that addition of IL-4 and IL-13 to CM resulted in the highest levels of CLEC5A, CD163, and CD206 and the greatest reduction in CD86, whereas CM or IL-4 and IL-13 alone led to modest changes (Figure 2, A and B, and Supplemental Figure 4D). To support generalizability beyond THP-1, we observed similar trends in primary human PBMC–derived MDMs through flow cytometry (Figure 2, C and D). These results indicate that CLEC5A was preferentially expressed in TAM-IM in vitro and could be stimulated with glioma CM, as well as IL-4 and IL-13.

To directly test whether *CLEC5A* modulates TAM polarization, we analyzed mRNA expression of TAM-IM (*CD163*, *IL10*, *MSR1*, *MRC1*, *CD16*, and *TGFB*) and TAM-INF (*CD86*, *iNOS*, *TNFA*, *CCL2*, *CCL5*, and *IL12p35*) markers in control, empty vector, sh-CLEC5A, and OE-CLEC5A groups using qPCR. All TAM-IM markers were downregulated in the sh-CLEC5A group and upregulated in the OE-CLEC5A group (Figure 2E). In contrast, there was no significant difference in TAM-INF biomarker expression among the experimental groups, with only *CD86*, *CCL5*, and *IL12p35* expression levels slightly increased in the OE-CLEC5A group (Figure 2F). To determine if these effects of *CLEC5A* on TAM polarization were similar in the presence of neoplastic cells, we cocultured GBM cells with THP-1 cells and analyzed cytokine secretion profiles. THP-1 cells were treated with PMA alone (control), IL-4 and IL-13 (positive control), shRNA negative control (sh-NC), sh-CLEC5A, OE empty vector (OE-NC), and OE-CLEC5A. The cytokine antibody microarray contained 20 cytokines (Supplemental Table 3). IL-1β, IL-4, IL-6, IL-8, IL-10, IL-13, and GM-CSF were significantly increased in the positive control and OE-CLEC5A groups compared with controls. In contrast, these cytokines were reduced in the sh-CLEC5A group (Figure 2G). Finally, in cocultures with patient-derived GBM neurospheres (NU02068), flow cytometry demonstrated that OE-CLEC5A macrophages had increased CLEC5A^+^CD163^+^ and CD206^+^ expression, with a reduction in CD86^+^ compared with the control, whereas CLEC5A knockdown produced the opposite changes (Figure 2, H and I). Collectively, these data indicate that CLEC5A induced polarization of TAMs toward an immunosuppressive phenotype.

### CLEC5A deletion in TAMs delays tumor progression by reprogramming the immunosuppressive TME.

**Figure 3:**
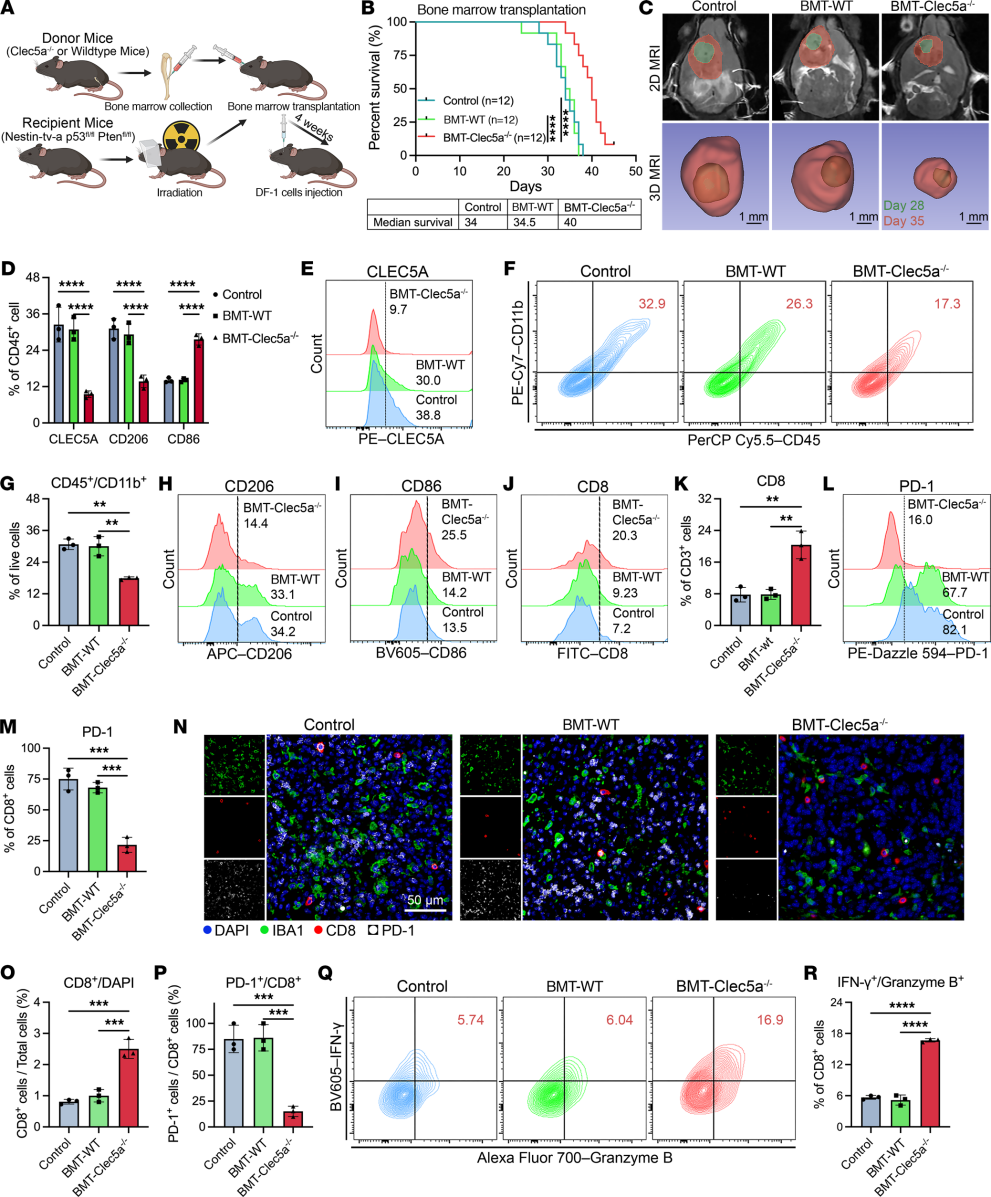
CLEC5A deletion in TAMs delays tumor progression by reprogramming the immunosuppressive TME. (**A**) Schematic diagram of the RCAS/tv-a mouse model showing procedures for bone marrow collection from donor mice, irradiation, BMT, and intracranial injection of DF-1 cells. (**B**) Kaplan-Meier survival curves for RCAS/tv-a mice in control, BMT-WT, and BMT-*Clec5a^–/–^* groups (*n* = 12 per group). (**C**) Representative 2D MRI monitoring and 3D reconstruction mapping depicting neoplastic growth of tumor-bearing mice 28 and 35 days following injection of DF-1 cells. Scale bars: 1 mm. (**D**) Histogram quantification showing the proportion of CLEC5A^+^, CD206^+^, and CD86^+^ cells among CD45^+^ cells (2-way ANOVA with Tukey’s multiple-comparison test). (**E**) Flow cytometry of digested mouse brain tissue from control, BMT-WT, and BMT-*Clec5a^–/–^* groups, showing the proportion of CLEC5A^+^ cells among CD45^+^ cells. (**F** and **G**) Representative flow cytometry plots and quantification of CD45^+^CD11b^+^ myeloid cells in mouse brain tumors across control, BMT-WT, and BMT-*Clec5a^–/–^* groups (1-way ANOVA with Tukey’s multiple-comparison test). (**H** and **I**) Flow cytometry of digested mouse brain tissue showing the proportion of CD206^+^ (**H**) and CD86^+^ (**I**) cells among CD45^+^ cells across control, BMT-WT, and BMT-*Clec5a^–/–^* groups. (**J** and **K**) Representative flow cytometry images and quantification of CD8^+^ cells in CD3^+^ T cells across the indicated groups (1-way ANOVA with Tukey’s multiple-comparison test). (**L** and **M**) Representative flow cytometry images and quantification of PD-1^+^ cells in CD8^+^ T cells across the indicated groups (1-way ANOVA with Tukey’s multiple-comparison test). (**N**) IF staining of mouse brain sections for IBA1, CD8, and PD-1 expression across control, BMT-WT, and BMT-*Clec5a^–/–^* groups. Scale bar: 50 μm. Original magnification, ×20. (**O** and **P**) Quantification of the proportion of CD8^+^ T cells within total cells (**O**) and PD-1^+^ cells among CD8^+^ T cells (**P**) by IF staining (1-way ANOVA with Tukey’s multiple-comparison test). (**Q** and **R**) Flow cytometry plots and histogram of digested mouse brain tissue showing the proportion of IFN-γ ^+^/granzyme B^+^ cells among CD8^+^ cells across the indicated groups (1-way ANOVA with Tukey’s multiple-comparison test). **P < 0.01, ***P < 0.001, and ****P < 0.0001.

To further uncover the effects of CLEC5A ablation in TAMs on their polarization and the TME immunosuppression in vivo, we utilized mice on a Nestin-tv-a p53^fl/fl^, Pten^fl/fl^ (NpP) background and induced tumor growth by intracranial injection of DF-1 cells (chicken fibroblasts) expressing RCAS-PDGFB-RFP and RCAS-Cre (RCAS/tv-a system). Recipient mice underwent adoptive bone marrow transplantation (BMT) from either *Clec5a*-WT (BMT-WT) or *Clec5a^–/–^* (BMT-*Clec5a^–/–^*) donors, alongside a control group without BMT (Figure 3A). Survival analysis revealed that mice receiving *Clec5a^–/–^* BMT had prolonged survival compared with mice in both the control and BMT-WT groups (Figure 3B). Tumor volume was assessed by MRI and quantified using 3D reconstruction, demonstrating no significant differences in tumor size among the 3 groups on day 28 following DF-1 cell injection. However, by day 35, tumor growth was markedly attenuated in the BMT-*Clec5a^–/–^* group compared with the control groups (Figure 3C and Supplemental Figure 5A). Given our prior findings that spontaneous necrosis typically emerges in the RCAS/tv-a GBM model during the 3–4 weeks after injection, we speculated that the onset of necrosis would lead to TAM recruitment into the TME in the timeframe, thereby accelerating tumor progression in controls, but not in BMT-*Clec5a^–/–^* mice (26). To assess the generalizability of these findings beyond the RCAS/tv-a model, we next evaluated the role of myeloid CLEC5A in an independent syngeneic CT-2A GBM model in immunocompetent C57BL/6 mice. Consistent with our findings in the RCAS/tv-a system, mice receiving BMT-*Clec5a^–/–^* had prolonged survival compared with BMT-WT controls (Supplemental Figure 5B). MRI-based volumetric analysis demonstrated reduced tumor burden and delayed tumor progression in the BMT-*Clec5a^–/–^* group (Supplemental Figure 5, C and D). We hypothesized that CLEC5A deficiency probably impairs TAM recruitment, inhibits polarization toward the TAM-IM phenotype, and alleviates T cell suppression, ultimately delaying tumor progression. To test this hypothesis, we collected postmortem brain tissues for paraffin embedment and IF staining, or processed fresh tissues for flow cytometric analysis (Supplemental Figure 5E).

We first assessed CLEC5A in CD45^+^ immune cells and found that its expression was significantly reduced in the BMT-*Clec5a^–/–^* group compared with both the control and BMT-WT groups (Figure 3, D and E), confirming efficient gene KO in the myeloid compartment. We then compared TAM infiltration and polarization across the 3 groups. Flow cytometric analysis revealed that mice in the BMT-*Clec5a^–/–^* group had a significantly lower proportion of CD45^+^CD11b^+^ TAMs among total cells (Figure 3, F and G). Furthermore, the percentage of CD206^+^ TAM-IM cells within the CD45^+^ cell population was markedly reduced (Figure 3, D and H), while the proportion of CD86^+^ TAM-INF cells was increased (Figure 3, D and I). Consistent with these findings, IF staining of mouse brain tissue confirmed reduced CLEC5A expression in the BMT-*Clec5a^–/–^* group, accompanied by decreased levels of IBA1^+^ and CD163^+^ myeloid cells (Supplemental Figure 5F). Together, these data suggest that CLEC5A contributes to both TAM recruitment and polarization toward the TAM-IM phenotype in vivo.

To further determine whether CLEC5A depletion modulates T cell responses and the immunosuppressive landscape of the TME, we next analyzed T cell infiltration and activation states. Flow cytometry revealed a significant increase in CD8^+^ cytotoxic T cell infiltration into tumors from BMT-*Clec5a^–/–^* mice compared with controls (Figure 3, J and K). Moreover, the proportion of programmed cell death 1–positive (PD-1^+^) exhausted cells within the CD8^+^ T cell population was markedly reduced (Figure 3, L and M). IF staining of mouse brain tissue also demonstrated increased infiltration of CD8^+^ T cells and a reduction in PD-1^+^ cells in the BMT-*Clec5a^–/–^* group, indicating a decrease in the population of exhausted T cells (Figure 3, N–P). Importantly, the percentages of IFN-γ^+^granzyme B^+^ activated cytotoxic CD8^+^ T cells were elevated (Figure 3, Q and R), indicating enhanced effector function. Collectively, these findings indicate that myeloid-specific CLEC5A deficiency delayed GBM progression and reprogrammed the TME, not only by reducing TAM infiltration and diminishing the immunosuppressive TAM-IM phenotype, but also by enhancing CD8^+^ T cell accumulation and effector function.

### PDPN directly binds CLEC5A and triggers TAM polarization.

**Figure 4:**
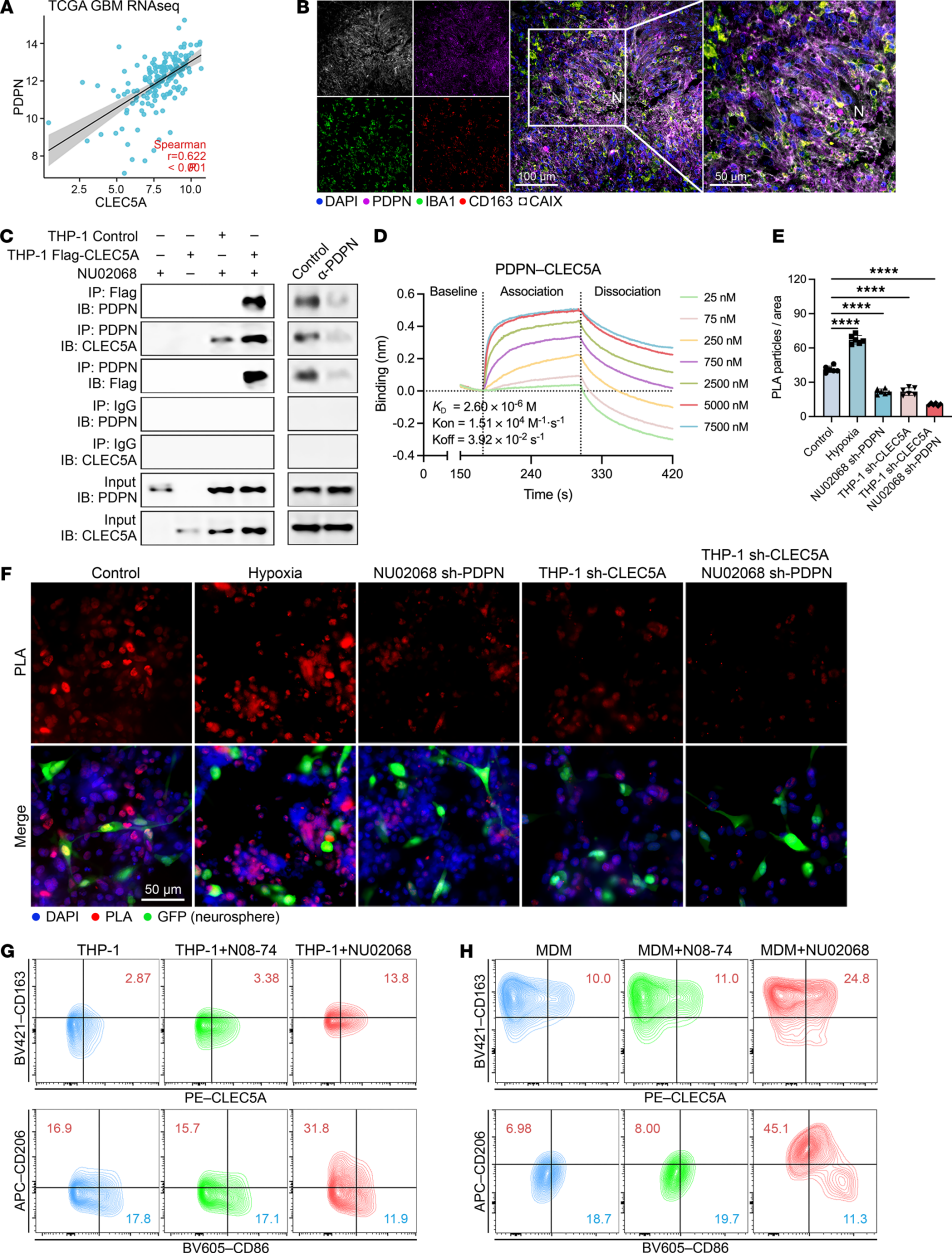
PDPN directly binds CLEC5A and triggers TAM polarization. (**A**) Spearman correlation analysis of *CLEC5A* and *PDPN* expression in TCGA datasets. (**B**) IF staining for CAIX (hypoxia), IBA1 (immune cell), CLEC5A, and PDPN in samples from patients with GBM. Scale bars: 100 μm and 50 μm (insets). N, necrosis. (**C**) Co-IP demonstrating binding of PDPN and CLEC5A in coculture of NU02068 neurospheres and Flag-tagged OE-CLEC5A THP-1 cells, using anti-PDPN–blocking antibody to show specificity. IB, immunoblot. (**D**) BLI analysis demonstrates direct cell-free binding of PDPN to CLEC2, with kinetic binding (Koff/Kon) and equilibrium affinity (*K_D_*) derived from global kinetic fitting. (**E** and **F**) Representative PLA images and quantification of PLA puncta showing endogenous PDPN-CLEC5A proximity in tumor-TAM cocultures under normoxia, hypoxia, and incorporation of sh-PDPN (tumor cell) and sh-CLEC5A (TAM) knockdowns (1-way ANOVA with Dunnett’s multiple-comparison test). Scale bar: 50 μm. (**G** and **H**) Representative flow cytometry plots for CLEC5A^+^CD163^+^, CD206^+^CD86^–^, and CD206^–^CD86^+^ in THP-1 cells (**G**) and MDMs (**H**) cocultured with high PDPN-expressing neurospheres (NU02068) or low PDPN-expressing neurospheres (N08-74). ****P < 0.0001.

To interrogate potential upstream mechanisms of CLEC5A activation in TAMs, we first compared the 3D structure of CLEC5A with other CLEC superfamily members with known activating ligands and found the greatest degree of similarity between CLEC2 and CLEC5A (Supplemental Figure 6, A and B). CLEC2 possesses a highly specific binding site for PDPN, which leads to its activation in multiple cell types, including platelets and megakaryocytes, B cells, myeloid cells, and DCs (27, 28). Since CLEC2 (*CLEC1B*) expression was evident at low levels or was absent in gliomas (Supplemental Figure 6C), we hypothesized that PDPN could be the activating ligand for TAM CLEC5A. To further explore this, we analyzed the relationship between *PDPN* with *CLEC5A* and *CLEC2* in GBMs based on TCGA and CGGA datasets, which showed a strong correlation between *PDPN* and *CLEC5A*, with no substantial correlation between *PDPN* and *CLEC2* (Figure 4A and Supplemental Figure 6, C and D). IF staining of samples from patients with GBM revealed high expression of both PDPN and CLEC5A in hypoxic perinecrotic regions, with proximity suggesting a potential physical interaction (Figure 4B). Ivy GAP and scRNA-seq analyses further demonstrated that *PDPN* was highly expressed in the perinecrotic zone and was enriched in mesenchymal-like (MES-like) hypoxia/major histocompatibility complex and MES-like, hypoxia-independent neoplastic programs (Supplemental Figure 3D and Supplemental Figure 6E). In addition, hypoxia module scores were associated with *PDPN* expression in neoplastic cells (Supplemental Figure 6F), supporting a hypoxia-linked context for PDPN expression and its potential interaction with CLEC5A in the perinecrotic niches. Furthermore, PDPN protein was variably expressed in patient-derived GBM neurospheres under normoxia, with increased expression observed under hypoxia (Supplemental Figure 6, G–J).

To determine if PDPN and CLEC5A were binding partners, we cocultured control THP-1 cells and THP-1 cells overexpressing Flag-CLEC5A with GBM cells (NU02068) and performed co-IP. We found that CLEC5A and PDPN could be co-immunoprecipitated using antibodies against CLEC5A, Flag-CLEC5A, or PDPN, indicating a protein-protein binding in vitro, and this interaction was reduced by a PDPN-blocking antibody (α-PDPN), confirming specificity (Figure 4C). To establish direct biochemical binding and obtain real-time association (Kon) and dissociation (Koff) kinetics and the equilibrium affinity (*K_D_*), we next performed biolayer interferometry (BLI) using purified proteins. As a positive control, PDPN-CLEC2 interaction showed robust binding kinetics (Kon = 8.68 × 10^5^ M^–1^ × s^–1^, Koff = 1.95 × 10^–2^ s^–1^) and an affinity of *K_D_* = 2.25 × 10^–8^ M, consistent with a previous report (29) (Supplemental Figure 6K). Under identical conditions, PDPN-CLEC5A exhibited concentration-dependent association and disassociation with a globally fitted Kon = 1.51 × 10^4^ M^–1^ × s^–1^ and Koff = 3.92 × 10^–2^ s^–1^, and an equilibrium *K_D_* = 2.60 × 10^–6^ M, supporting direct binding between PDPN and CLEC5A (Figure 4D). To further validate this interaction in situ, we performed proximity ligation assays (PLAs) in tumor-macrophage cocultures. PLA revealed discrete PDPN-CLEC5A puncta that were significantly increased under hypoxic conditions compared with normoxia. Importantly, genetic knockdown of either PDPN in tumor cells or CLEC5A in macrophages markedly reduced PLA signal, confirming the specificity of the interaction (Figure 4, E and F). Similar hypoxia-enhanced PDPN-CLEC5A proximity was observed in primary human PBMC–derived MDMs cocultured with GBM neurospheres (Supplemental Figure 6, L and M).

We next cocultured activated THP-1 cells with normoxic GBM neurospheres with high PDPN expression (NU02068) or low PDPN expression (N08-74) to further determine whether PDPN activates CLEC5A and leads to TAM-IM polarization. Flow cytometry indicated that THP-1 cells cocultured with NU02068 had a markedly higher proportion of CLEC5A^+^CD163^+^ macrophages, enhanced CD206 expression, and reduced CD86 expression compared with those cocultured with N08-74 (Figure 4G and Supplemental Figure 6N). Similar trends were observed in primary human PBMC–derived MDMs cocultured with these 2 human-derived primary neurospheres (Figure 4H and Supplemental Figure 6O). Collectively, these findings suggest that PDPN is an activating ligand for CLEC5A in the hypoxic perinecrotic zone of GBM, contributing to TAM-IM polarization.

### Tumor-derived PDPN promotes TAM-IM polarization through CLEC5A in vivo.

**Figure 5:**
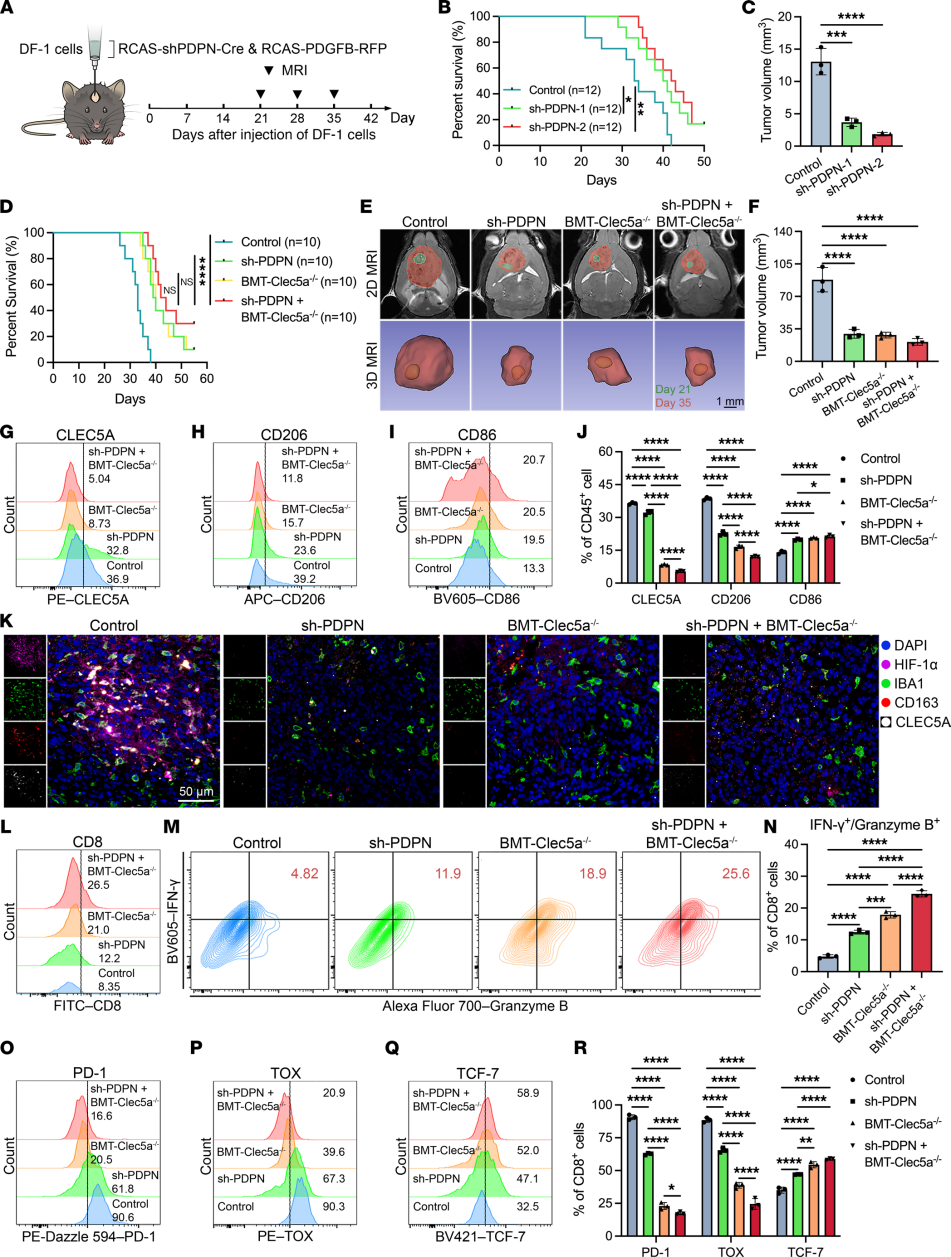
Tumor-derived PDPN promotes TAM-IM polarization through CLEC5A in vivo. (**A**) Schematic diagram of in vivo experimental design using the RCAS/tv-a mouse model to evaluate the effects of sh-PDPN in glioma cells. (**B**) Kaplan-Meier survival curves for RCAS/tv-a mice treated with sh-PDPNs (*n* = 12 per group). (**C**) Histogram showing tumor volumes in mice in control, sh-PDPN-1, and sh–PDPN-2 groups 28 days after injection of DF-1 cells (1-way ANOVA, Dunnett’s multiple-comparisons test). (**D**) Kaplan-Meier survival analysis of tumor-bearing mice in the RCAS/tv-a, PDGFB-driven glioma model with control, tumor cell PDPN knockdown (sh-PDPN), BMT-*Clec5a^–/–^*, or combined sh-PDPN plus BMT-*Clec5a^–/–^* (*n* = 10 per group). (**E**) Representative 2D MRI images (top) and corresponding 3D tumor reconstructions (bottom) from each group at day 21 and day 35 time points. Scale bar: 1 mm. (**F**) Quantification of tumor volume across the groups shown in **D** and **E** (1-way ANOVA with Tukey’s multiple-comparison test). (**G**–**I**) Representative flow cytometry images showing the proportion of CLEC5A (**G**), CD206 (**H**), and CD86 (**I**) in tumor-infiltrating myeloid cells across the indicated groups. (**J**) Quantification of CLEC5A, CD206, and CD86 expression within CD45^+^ myeloid cells across groups (2-way ANOVA with Tukey’s multiple-comparison test). (**K**) IF staining for HIF-1α, IBA1, CD163, and CLEC5A in mouse brain sections. Scale bar: 50 μm. Original magnification, ×20. (**L**) Representative flow cytometry images of CD8^+^ cells in CD3^+^ T cells across groups. (**M** and **N**) Representative flow cytometry plots and quantification of IFN-γ^+^granzyme B^+^ expression within CD8^+^ T cells across groups (1-way ANOVA, Tukey’s multiple-comparisons test). (**O**–**Q**) Representative images of PD-1 (**O**), TOX (**P**) (terminal exhausted), and TCF-7 (**Q**) (precursor) expression in CD8^+^ T cells across groups. (**R**) Quantification of PD-1, TOX, and TCF-7 expression in CD8^+^ T cells (2-way ANOVA with Tukey’s multiple-comparison test). *P < 0.05, **P < 0.01, ***P < 0.001, and ****P < 0.0001.

To further investigate the role of tumor-derived PDPN as an activator of TAM CLEC5A in GBM, we constructed an RCAS vector containing sh-PDPN to knock down PDPN expression in glioma cells using the RCAS/tv-a system (Figure 5A). Tumor burden was assessed using MRI-based volumetry and 3D reconstruction. PDPN knockdown significantly reduced tumor growth and prolonged survival of mice compared with control tumors (Figure 5, B and C, and Supplemental Figure 7A). To validate the knockdown efficiency of sh-RNAs, we dissociated and digested mouse brain tumor tissue, which showed that sh-PDPN significantly reduced the proportion of PDPN^+^ cells among CD45^–^CD11b^–^ non-myeloid cells (Supplemental Figure 7, C and D). IF staining of mouse brain tissue also revealed that sh-PDPN treatment reduced PDPN expression, as well as the expression of phosphorylated STAT3 (p-STAT3) and CD133 (Supplemental Figure 7B). Additionally, sh-PDPN not only decreased the proportion of CD45^+^CD11b^+^ myeloid cells in mouse brain tumor tissues, but also reduced the proportion of CD206^+^ cells and increased CD86^+^ cells among CD45^+^ cells, indicating that sh-PDPN diminished TAM infiltration and attenuated the polarization toward the TAM-IM phenotype (Supplemental Figure 7, E–G).

To determine whether the antitumor effects of PDPN knockdown are mediated through myeloid CLEC5A, we next performed a genetic epistasis experiment by combining tumor cell–specific PDPN knockdown with BMT-*Clec5a^–/–^* in our PDGFB-driven RCAS/tv-a model. If PDPN knockdown exerts its antitumor effects primarily through CLE5A signaling, then the benefit of sh-PDPN or BMT-*Clec5a^–/–^* alone should be attenuated, or at least nonadditive, in mice treated with sh-PDPN combined with BMT-*Clec5a^–/–^*. We found that the combination of sh-PDPN plus BMT-*Clec5a^–/–^* did not result in significantly additional improvement of survival relative to sh-PDPN or BMT-*Clec5a^–/–^* alone (Figure 5D). Similarly, tumor burden analysis by MRI and 3D reconstruction showed no further reductions in the combined group compared with either intervention alone (Figure 5, E and F), indicating that the therapeutic effect of PDPN knockdown was dependent on myeloid CLEC5A in vivo.

At the cellular level, flow cytometric analysis revealed that the combination group exhibited a reduced proportion of CD45^+^CD11b^+^ myeloid cells compared with the control and sh-PDPN groups, whereas no significant difference was observed between the BMT-*Clec5a^–/–^* and combined groups (Supplemental Figure 7, H and I). In addition, the proportions of CLEC5A^+^ and CD206^+^ cells were reduced in both the sh-PDPN and BMT-*Clec5a^–/–^* groups and reached the lowest levels in the combination group, whereas no significant difference in CD86^+^ cell was found across the single-intervention groups and the combination group (Figure 5, G–J). Consistent with these findings, IF staining showed that the proportions of IBA1^+^ cells among total cells and CD163^+^ cells within IBA1^+^ cells were significantly decreased in the combination group compared with the control group but were slightly decreased compared with the sh-PDPN group and comparable to the BMT-*Clec5a^–/–^* group (Figure 5K and Supplemental Figure 7, L and M).

Given the central role of TAMs in shaping T cell immunity, we next assessed CD8^+^ T cell responses. Flow cytometry showed increased CD8^+^ T cell infiltration in both the sh-PDPN and BMT-*Clec5a^–/–^* groups, with the highest levels in the combination group (Figure 5L and Supplemental Figure 7P). Functional profiling further demonstrated enhanced IFN-γ^+^granzyme B^+^ cytotoxic CD8^+^ T cells (Figure 5, M and N). In parallel, T cell exhaustion markers PD-1 and thymocyte selection–associated high mobility group box protein (TOX) were diminished, whereas T cell factor 7 (TCF-7) progenitor–exhausted (PEX) populations were increased in the combination group relative to the control group, whereas only slight differences were noted between the BMT-*Clec5a^–/–^* and combination groups (Figure 5, O–R). These finding were also supported by IF staining, which showed increased CD8^+^ T cell infiltration and reduced PD-1^+^ cells within tumors from all experimental arms compared with controls, while no significant differences were observed across the single intervention groups and the combination group (Supplemental Figure 7, N O, and Q). Taken together, these findings indicate that the antitumor and immunomodulatory effects of PDPN knockdown are, at least in part, dependent on myeloid CLEC5A signaling in vivo, supporting a model in which tumor-derived PDPN acts upstream of CLEC5A to drive TAM-IM polarization and immunosuppressive TME remodeling.

### CLEC5A promotes TAM-IM polarization and immunosuppressive TME through Syk/STAT3 signaling.

**Figure 6:**
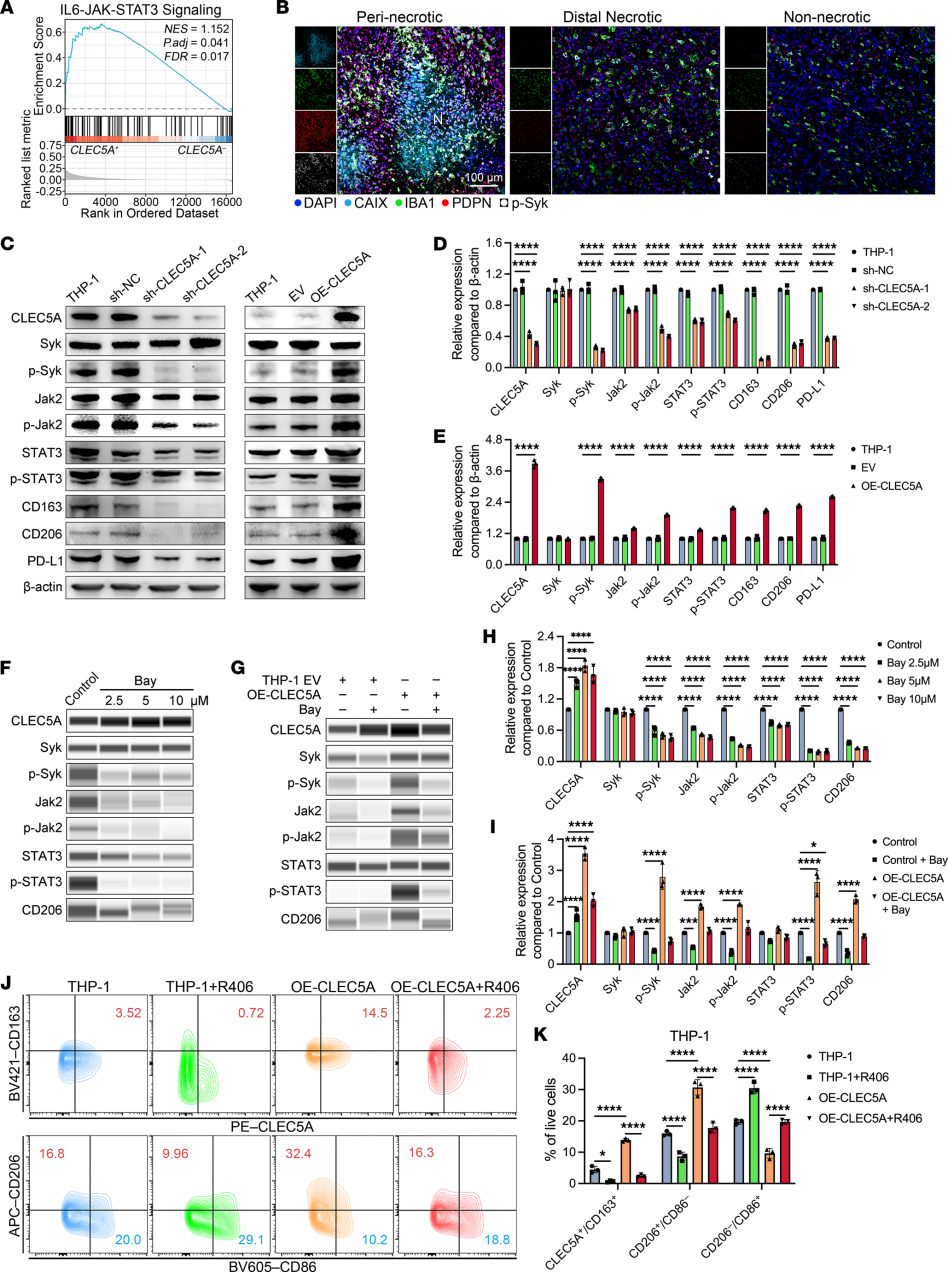
CLEC5A promotes TAM-IM polarization and immunosuppressive TME through Syk/STAT3 signaling. (**A**) GSEA showing that *CLEC5A^+^* TAM-IM are enriched in IL-6/JAK/STAT3 signaling using scRNA-seq data (NES = 1.152, *P*.adj = 0.041). (**B**) IF staining for CAIX, IBA1, PDPN, and p-Syk performed on perinecrotic and distal necrotic regions in necrotic GBM, as well as non-necrotic regions in non-necrotic tumor. Scale bar: 100 μm. Original magnification, ×20. (**C**) Western blot showing protein expression of CLEC5A, Syk, p-Syk, Jak2, p-Jak2, STAT3, p-STAT3, CD163, CD206, and PD-L1 in THP-1 cells after CLEC5A knockdown or overexpression. (**D** and **E**) Relative levels of these proteins compared with those of β-actin in **C** (2-way ANOVA with Dunnett’s multiple-comparison test). (**F**) Simple Western (Jess) protein analysis of CLEC5A, Syk, p-Syk, Jak2, p-Jak2, STAT3, p-STAT3, and CD206 following treatment with Bay (2.5, 5, and 10 μM). (**G**) Simple Western (Jess) protein analysis of Bay-treated THP-1 cells (control and OE-CLEC5A) expressing the proteins CLEC5A, Syk, p-Syk, Jak2, p-Jak2, STAT3, p-STAT3, and CD206. (**H** and **I**) Relative protein levels of the proteins in **F** and **G** (2-way ANOVA with Dunnett’s multiple-comparison test). (**J** and **K**) Representative flow cytometry plots and quantification of TAM polarization markers in THP-1 cells following treatment with the Syk inhibitor R406 in the presence or absence of OE-CLEC5A (2-way ANOVA with Tukey’s multiple-comparison test). *P < 0.05 and ****P < 0.0001.

To investigate downstream signaling mechanisms by which CLEC5A promotes TAM polarization, we began by analyzing RNA-seq and scRNA-seq data. Gene set enrichment analysis (GSEA) showed that CLEC5A-associated genes were enriched in the IL-6/JAK/STAT3 signaling pathway based on both TCGA data (NES = 2.680, *P*.adj = 0.005, Supplemental Figure 8A) and scRNA-seq data (NES = 1.152, *P*.adj = 0.041, Figure 6A), suggesting that CLEC5A could potentially mediate it effects on TAMs through JAK/STAT pathways. Since previous studies have indicated that CLEC5A activates spleen-associated tyrosine kinase (Syk) in inflammatory diseases and autoimmune disorders (15, 30) and have also suggested a link between Syk signaling and STAT3 activation (31, 32), we examined downstream signaling pathways that include Syk and STAT3. Chord diagram analysis found a strong positive correlation between *CLEC5A* and Syk signaling molecules (*Syk*, *STAT3*), as well as immunosuppression-related factors (*MRC1*, *MSR1*, and *CD274*) (Supplemental Figure 8, B and C).

To validate these findings and further link PDPN to Syk activation, we performed IF staining of human GBM specimens and demonstrated spatial colocalization of PDPN^+^ tumor cells, CLEC5A^+^ TAMs, and p-Syk activation within perinecrotic niches (Figure 1, K and L, Figure 6B, and Supplemental Figure 3, G and H). Notably, we observed that p-Syk^+^IBA1^+^ cells were enriched in CAIX^+^ necrotic niches, with diminishing density in CAIX^–^ regions more distal from necrosis and in non-necrotic tumors (Figure 6B and Supplemental Figure 8D), supporting in situ activation of this signaling axis. At the tissue level, IF staining analysis of mouse brain tissue showed that either sh-PDPN or BMT-*Clec5a^–/–^* was associated with reduced p-Syk expression in IBA^+^ myeloid cells, whereas the combination of sh-PDPN and BMT-*Clec5a^–/–^* did not result in an additional reduction of p-Syk expression (Supplemental Figure 8, E and F). Next, we examined the effects of CLEC5A expression by THP-1 cells on downstream signaling elements. Knockdown of CLEC5A significantly downregulated the expression of p-Syk, Jak2, p-Jak2, STAT3, p-STAT3, CD163, CD206, and PD-L1, while overexpression of CLEC5A significantly upregulated the expression of these proteins (Figure 6, C–E). Given the lack of credible CLEC5A pharmacologic inhibitors, we introduced 2 highly selective Syk inhibitors, Bay 61-3606 (Bay) and R406, to determine whether CLEC5A mediates TAM-IM polarization through Syk/Jak2/STAT3 signaling in vitro (33, 34). Protein analysis by Simple Western (Jess) showed that Bay had a dose-dependent effect on reducing the expression of p-Syk, Jak2, p-Jak2, STAT3, p-STAT3, and CD206 (Figure 6, F and H). Importantly, Syk inhibition partially rescued the increased expression of these proteins caused by CLEC5A overexpression (Figure 6, G and I). Flow cytometry further indicated that CLEC5A overexpression increased the proportion of CLEC5A^+^CD163^+^ cells and CD206 expression, while reducing CD86 expression in THP-1 cells, and these effects were restored by Syk inhibition (Figure 6, J and K). Taken together, these findings indicate that PDPN-CLEC5A activated Syk/Jak2/STAT3 signaling in vitro, which was associated with a TAM-IM polarization and immunosuppressive programming.

### Syk inhibition suppresses tumor progression and reprograms the immunosuppressive TME in vivo.

**Figure 7:**
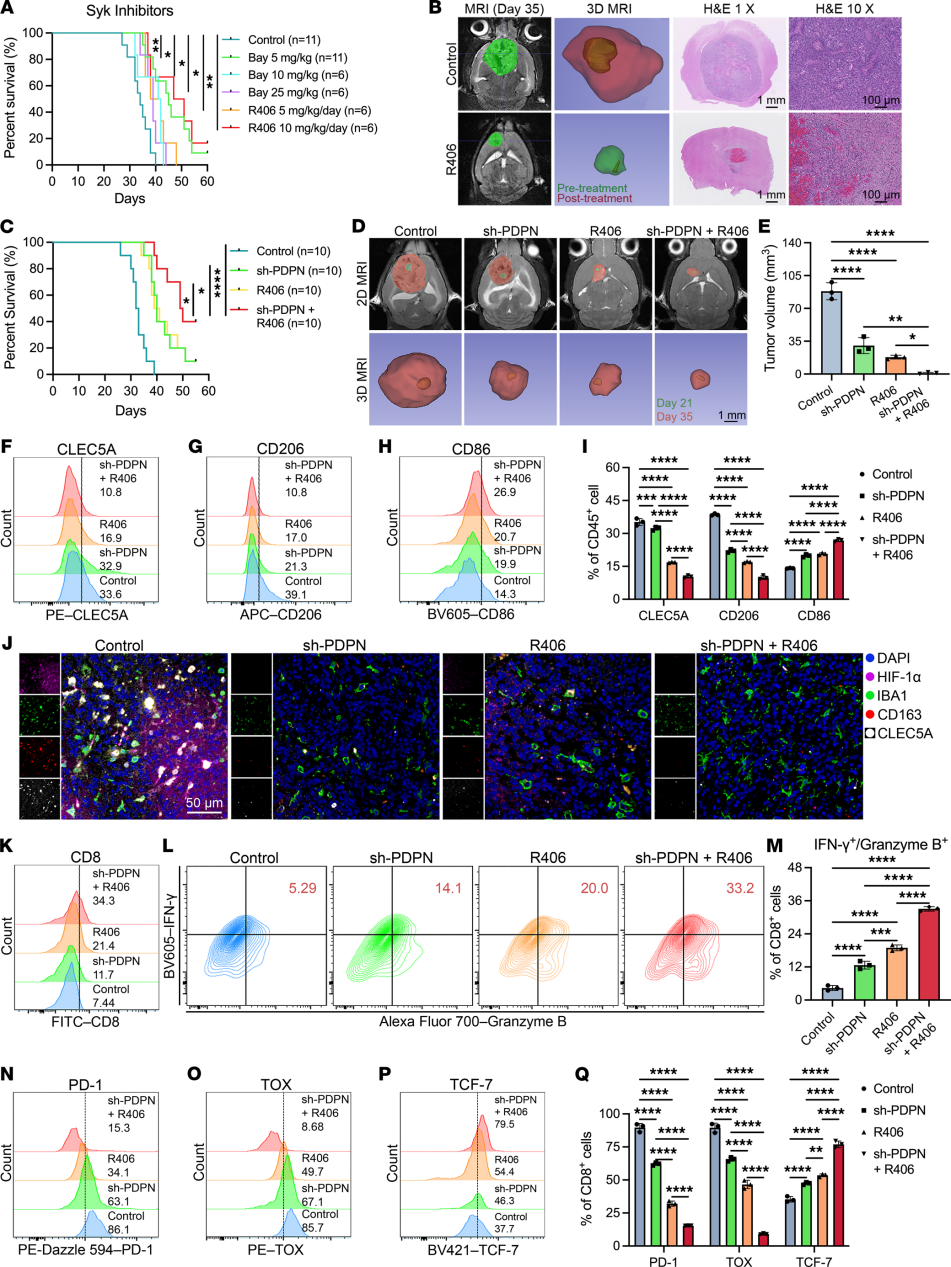
Syk inhibition suppresses tumor progression and reprograms the immunosuppressive TME in vivo. (**A**) Kaplan-Meier survival curves for RCAS/tv-a mice treated with Bay or R406. (**B**) MRI and H&E-stained images showing tumor size and histological features in control and R406-treated mice. Scale bars: 1 mm and 100 μm. (**C**) Kaplan-Meier survival analysis of tumor-bearing mice treated with control, sh-PDPN, the Syk inhibitor R406, or a combination of sh-PDPN plus R406 (*n* = 10 per group). (**D**) Representative 2D MRI images (top) and corresponding 3D tumor reconstructions (bottom) from mice of each treatment group. Scale bar: 1 mm. (**E**) Quantification of tumor volume across the groups shown in **C** and **D** (1-way ANOVA with Tukey’s multiple-comparison test). (**F**–**H**) Representative flow cytometry images showing the proportion of CLEC5A (**F**), CD206 (**G**), and CD86 (**H**) in tumor-infiltrating myeloid cells across the indicated groups. (**I**) Quantification of CLEC5A, CD206, and CD86 expression in CD45^+^ myeloid cells across groups (2-way ANOVA with Tukey’s multiple-comparison test). (**J**) IF staining of HIF-1α, IBA1, CD163, and CLEC5A in mouse brain sections. Scale bar: 50 μm. Original magnification, ×20. (**K**) Representative flow cytometry plots of CD8^+^ expression among CD3^+^ T cells across groups. (**L** and **M**) Representative flow cytometry plots and quantification of IFN-γ^+^granzyme B^+^ expression within CD8^+^ T cells across groups (1-way ANOVA with Tukey’s multiple-comparison test). (**N**–**P**) Representative plots of PD-1 (**N**), TOX (**O**) (terminal exhausted), and TCF-7 (**P**) (precursor) expression in CD8^+^ T cells across groups. (**Q**) Quantification of PD-1, TOX, and TCF-7 expression in CD8^+^ T cells (2-way ANOVA with Tukey’s multiple-comparison test). *P < 0.05, **P < 0.01, ***P < 0.001, and ****P < 0.0001.

To examine the effects of pharmacological Syk inhibition on glioma growth, we treated mice bearing PDGFB-driven GBMs in the RCAS/tv-a model with the Syk inhibitors Bay (i.p. at 5, 10, and 25 mg/kg/day) and R406 (i.p. at 5 and 10 mg/kg/day) (Supplemental Figure 8G). Survival analysis showed that mice treated with either Bay or R406 survived longer than did the control mice, and those treated with Bay at 5 mg/kg/day or R406 at 10 mg/kg/day showed the greatest therapeutic response and survived the longest within their treatment groups, respectively (Figure 7A). Given that R406 is the active metabolite of fostamatinib — an FDA-approved drug for chronic immune thrombocytopenia in adults — we selected R406 for subsequent analyses (35). MRI-based volumetry and 3D reconstruction mapping and corresponding tissue sampling showed that R406 significantly suppressed glioma growth by day 35 (Figure 7B and Supplemental Figure 8H). Histopathological analysis also showed decreased necrosis and “pseudopalisading” structures in the treatment groups (Figure 7B). IF staining of mouse brain sections revealed that R406 reduced p-Syk and p-STAT3 expression in IBA1^+^ cells and led to a lower ratio of CD163^+^/IBA1^+^ cells, indicating that Syk inhibition reduced the infiltration of TAM-IM (Supplemental Figure 8, I–K). Flow cytometry showed that R406 reduced the infiltration of CD11b^+^CD45^+^ TAMs as well as the percentage of the CD206^+^ cells among CD45^+^ cells (Supplemental Figure 8, L and M). Overall, these findings indicate that Syk inhibition suppressed glioma growth, inhibited Syk phosphorylation and activation, reduced TAM infiltration, and suppressed the polarization of TAMs toward the TAM-IM phenotype.

To further assess pathway dependency and therapeutic potential, we combined tumor-intrinsic PDPN knockdown with pharmacologic Syk inhibition (R406) in vivo. We conducted a 4-arm experiment, including control, sh-PDPN, R406 (5 mg/kg/day), and combined sh-PDPN plus R406 groups. Survival analysis demonstrated that the combination group exhibited the most pronounced survival benefit compared with control group and each single-treatment group (Figure 7C). MRI-based volumetric analysis and 3D tumor reconstruction revealed that the combination treatment resulted in the greatest suppression of tumor growth, with significantly smaller tumor volumes compared with both the sh-PDPN and R406 single-intervention groups (Figure 7, D and E). These findings indicate that dual targeting of PDPN and Syk provided enhanced suppression of tumor progression in vivo.

To uncover the effects of the dual treatment (sh-PDPN plus R406) on TAM infiltration and polarization, we conducted flow cytometry and found that the combination group further reduced the proportion of CD45^+^/CD11b^+^ myeloid cells compared with single interventions (Supplemental Figure 9, A and B). Moreover, the percentage of CLEC5A^+^ and CD206^+^ TAM-IMs was decreased, whereas CD86^+^ TAM-INFs were relatively increased in the combination group (Figure 7, F–I), indicating a stronger shift away from an immunosuppressive macrophage phenotype. Consistently, IF staining of mouse brain tissue also showed that PDPN knockdown combined with Syk inhibition significantly reduced IBA1^+^ myeloid cell infiltration and diminished the proportion of CD163^+^ and p-Syk^+^ cells within IBA1^+^ myeloid cells (Figure 7J, Supplemental Figure 7, L and M, Supplemental Figure 8F, and Supplemental Figure 9E).

To further investigate the effect of PDPN knockdown combined with Syk inhibition on the immunosuppressive TME, we assessed CD8^+^ T cell infiltration and functional activation. Flow cytometric analysis of dissociated tumor tissues confirmed a higher proportion of CD8^+^ cytotoxic T cells among CD3^+^ total T cells in the combination group compared with the control and single-intervention groups (Figure 7K and Supplemental Figure 9F). Notably, the percentage of IFN-γ^+^granzyme B^+^ cells among CD8^+^ T cells was significantly elevated in the combination group (Figure 7, L and M), indicating enhanced activation. In addition, markers of T cell terminal exhaustion, including PD-1 and TOX, were reduced, while TCF-7^+^ PEX T cells were significantly increased in the combination group compared with the control and single-intervention groups (Figure 7, N–Q), consistent with improved T cell fitness and activation. IF staining of mouse brain sections further indicated that the combination treatment increased the proportion of CD8^+^ cells among total cells but resulted in the lowest level of PD-1^+^ cells within CD8^+^ T cells (Supplemental Figure 7, N and O, and Supplemental Figure 9G). Given the well-documented sex-dimorphic immune responses (36), we included both male and female mice at a ratio of approximately 1:1. No significant sex-dependent differences were observed in the measured outcomes in this study (Supplemental Figure 9, H–K). Taken together, these data suggest that combined targeting of upstream (PDPN) and downstream (Syk) components of this pathway, the PDPN/CLEC5A/Syk signaling axis, not only delayed tumor progression but also more effectively reprogrammed the TME, reducing TAM-mediated immunosuppression and promoting cytotoxic T cell responses. These findings are consistent with PDPN and CLEC5A-Syk functioning within a shared signaling axis and suggest that dual targeting may provide a more effective therapeutic strategy than either intervention alone in GBM.

## Discussion

The GBM TME is highly immunosuppressive, a property largely propagated through TAM influx and reprogramming, as GBM exhibits one of the strongest TAM signatures and highest TAM/lymphocyte ratios among all solid tumors (37). TME properties are tightly coupled with the development of necrosis, which has been recognized as a poor prognostic feature among gliomas for over a century and heralds a rapid growth phase characterized by accelerated radial expansion (3). Necrosis is accompanied by severe hypoxia in perinecrotic zones, and HIF-1 and HIF-2 transcriptional programs, among others, contribute to the adaptive response but also modulate immune reactions (38). The massive influx of TAMs from the vasculature in GBM is initiated as a sterile inflammatory response to necrosis and leads to a high TAM density in the perinecrotic niche, where they are heavily influenced by glioma cells, especially GSCs that are also enriched in the hypoxic zone (3, 6, 26). Our findings reveal that CLEC5A was highly upregulated by TAMs in hypoxic, perinecrotic regions of GBM, where it was activated by PDPN expressed by glioma cells, leading to TAM polarization to an immunosuppressive phenotype by signaling through the PDPN/CLEC5A/Syk/JAK/STAT3 axis.

Since the protumorigenic and immunosuppressive properties of TAMs are widely recognized, a common theme in neuro-oncology research has been to reverse the polarization, both to induce innate and adaptive immunogenicity and to promote immunotherapy efficacy (39, 40). Prior studies have uncovered numerous markers of the immunosuppressive phenotype, including *CD163*, *MRC1* (*CD206*), *MSR1* (*CD204*), *IL10*, *CD64*, and *TGFB*, yet specific mechanisms related to these markers have not fully matured to the point of successful therapeutic targeting for reversal (41, 42). Many factors emerge within the evolving GBM TME that strongly influence TAM phenotypes, including hypoxia, necrosis, cytokine signaling, and metabolic stress. The development of necrosis reorders the TME, with the hypoxic niche at its center attracting TAM-IMs and excluding cytotoxic T lymphocytes by factors including CCL8 and IL-1β (4, 43). IL-10 and TGF-β also promote TAM polarization toward protumoral functions by downregulating proinflammatory responses and facilitating tissue remodeling (8). Metabolic perturbations, including those involving fatty acids and cholesterol influence TAM function, redirecting the TAMs to a state that supports glioma cell proliferation and survival (44, 45).

The current findings add to these mechanisms by demonstrating that CLEC5A is a TAM surface receptor that binds to PDPN and signals through Syk-dependent signaling in a manner that can be reversed pharmacologically. Previous work has shown that CLEC5A expressed by myeloid cells phosphorylates DAP12 to secrete cytokines and chemokines such as TNF-α, IL-8, IL-1β, IL-6, IL-17A, CCL2, CCL3, and CCL22 that regulate immune responses in a variety of inflammatory responses (14, 15, 30), and most of these cytokines and chemokines have been linked to an immunosuppressive TME, GSC enrichment, and glioma progression (46, 47). However, CLEC5A’s specific role in regulating these mechanisms in GBM has not been defined. Our findings suggest that CLEC5A expression levels increase with the hypoxic gradient that develops as GBM transitions from a non-necrotic to a necrotic growth phase and are associated with enhanced myeloid cell infiltration into the perinecrotic regions. Mice receiving BMT from *Clec5a*-deficient donors showed prolonged survival, reduced accumulation of TAM-IMs and exhausted T cells, and increased infiltration of cytotoxic T cells, underscoring the critical role of CLEC5A in shaping the immunosuppressive TME in GBM. Once within the perinecrotic niche, TAMs encounter highly hypoxic glioma cells expressing PDPN, which binds to and activates CLEC5A signaling (3, 4).

We further identify PDPN as an activating ligand of CLEC5A on the basis of insights gained from structural similarities between CLEC5A and the CLEC family member CLEC2, a known receptor for PDPN on platelets (48). PDPN is a marker of poor prognosis in patients with GBM, yet its loss leads to only modest effects on glioma growth and survival in mouse models (49). Others have indicated that PDPN may confer resistance to radiotherapy or facilitate immune evasion and TAM polarization (19, 21, 50). Here, we have shown that the interaction between PDPN and CLEC5A activated downstream signaling pathways, enhancing the secretion of immunosuppressive cytokines such as IL-1β, IL-6, IL-10, and TGF-β, which in turn increased the expression of PDPN in GBM cells. This positive feedback loop could potentially sustain the immunosuppressive TME that facilitates tumor progression.

Pharmacologic inhibition of Syk, as well as genetic silencing of CLEC5A in TAMs and PDPN in glioma cells, reduced TAM infiltration and immunosuppressive polarization, and increased cytotoxic activation T cell accumulation in our in vivo models. These interventions led to delayed tumor growth, prolonged survival, and reversed TME immunosuppression in GBM-bearing mice, suggesting that targeting the PDPN/CLEC5A/Syk axis holds promise as a potential therapeutic approach for GBM. In particular, the Syk inhibitor R406, the active metabolite of fostamatinib, has immunomodulatory and tumor-suppressive properties and is being used successfully to treat patients with hematologic malignancies and autoimmune disorders (33, 34, 51). In addition to its effects on myeloid signaling, prior studies suggest that Syk inhibition may also exert tumor cell–intrinsic effects, including modulation of proliferation, migration, and invasion of GSCs and survival signaling in GBM (33, 34). While our data primarily support a role for R406 in reprogramming the immune microenvironment, we cannot exclude potential direct effects on glioma cells, which may contribute to the observed therapeutic benefit and warrant further clinical investigation.

Finally, as sex differences have been increasingly recognized in GBM biology and immune responses (36), we considered the potential effect of biological sex in our in vivo models. Although our study was not powered to detect sex-specific effects, both male and female mice were included in the RCAS/tv-a experiments, and no consistent sex-dependent differences in survival were observed (Supplemental Figure 9, H–K). Future studies with stratified designs will be important to more fully assess potential sex-specific modulation of the PDPN/CLEC5A/Syk axis and its therapeutic targeting.

## Methods

### Sex as a biological variable.

Sex was not considered as a biological variable in this study. Both male and female mice were included in the animal experiments at a ratio of approximately 1:1. No sex-based stratification or subgroup analyses were performed.

Additional details on methods are provided in the Supplemental Methods.

### scRNA-seq analysis.

scRNA-seq data were processed and analyzed using the Seurat package in R. First, we imported the scRNA-seq data and created a Seurat object through the CreateSeuratObject algorithm. Cells with an nCount_RNA value of greater than 8,000 or of less than 200, or cells with a percent.mt value greater than 20% were filtered out for quality control, and data were then standardized using the LogNormalize method. Principal component analysis linear dimensionality reduction and uniform manifold approximation and projection (UMAP) nonlinear dimensionality reduction were used to cluster similar cells. Next, we performed the FindMarkers algorithm to determine each cluster’s differential expression markers. We first distinguished clusters of malignant cells from nonmalignant cells using *GAP43*, *GPM6B*, *PTN*, *GFAP*, *SOX2*, *OLIG2*, *CD44*, *CCND2*, and *PARP1* (24). Then, *AIF*, *CD68*, *CD163*, and *MSR1* identified TAM-IM, whereas clusters with *AIF*, *CD68*, *CD86*, and *CD163*^lo^ were regarded as TAM-INF. *CD3G* cells were considered T cells. We then calculated the number and proportion of the various cell types identified. Next, we analyzed the data using an established hypoxia gene set, MSigDB Hallmark Hypoxia (Hypoxia_HM), and the Ucell package. Scores were computed after stringent quality control and are displayed on the refined UMAPs. Correlation analyses were performed to test the association between the hypoxia module score and CLEC5A and PDPN expression.

### IF staining.

Human GBM tissues, mouse brain sections, and THP-1 cells were incubated with primary antibodies (Supplemental Table 4) overnight. IF targets were detected with Alexa Fluor–labeled anti-rabbit, anti-mouse, anti-rat, and anti-chicken secondary antibodies (1:500, A-11029, A-21434, A-21449 and A-21039, Invitrogen, Thermo Fisher Scientific). Images were obtained using Olympbus and ZEISS microscopes.

### Western blots and Simple Western (Jess) analysis.

Tissues and cells were lysed in RIPA buffer (89900, Thermo Fisher Scientific) supplemented with protease inhibitor (1:100, 78430, Thermo Fisher Scientific) and phosphatase inhibitor (1:100, 78428, Thermo Fisher Scientific). After a 30-minute incubation on ice, the total protein concentration was determined using the DC protein assay (5000111, Bio-Rad) according to the manufacturer’s protocol. Western blot analysis was conducted as previously described (52). Briefly, 30 μg protein was loaded per well and then subjected to SDS-PAGE. Next, proteins were transferred onto PVDF membranes (ISEQ00010, MilliporeSigma) and blocked with 5% milk. Then, PVDF membranes were incubated with primary antibodies (Supplemental Table 4) overnight at 4°C. Protein bands were detected using goat anti–rabbit or anti–mouse IgG secondary antibodies (1:3,000, ZB-2301, ZB-2305, ZSGB-Bio) for 1 hour at room temperature. Protein expression was analyzed using chemiluminescence (WBKLS0500, MilliporeSigma) via GBOX (Syngene). Each experiment was performed independently 3 times.

We also utilized the Simple Western (Jess) system, which automates protein separation and immunodetection of traditional Western blotting. The Jess and Protein Normalization Module (DM-PN02, Bio-Techne) were used to detect the expression of PDPN in different neurospheres as well as CLEC5A, Syk, p-Syk, Jak2, p-Jak2, STAT3, p-STAT3, and CD206, according to the manufacturer’s protocol (ProteinSimple, Bio-Techne).

### Flow cytometry.

To investigate the effect of CLEC5A on TAM polarization and the immunosuppressive TME in GBM through flow cytometry, activated THP-1 cells (control, empty vector, and OE-CLEC5A) were cocultured with NU02068 neurospheres for 48 hours and then digested with Accutase Cell Dissociation Reagent (A1110501, Gibco, Thermo Fisher Scientific). Also, mouse brain tissues were digested using the NeuroCult Enzymatic Dissociation Kit (05715, STEMCELL Technologies) according to the manufacturer’s protocol. Next, cells were collected and washed with FACS buffer (PBS + 1%) 3 times, stained with the Zombie Aqua Fixable Viability Kit (423101, BioLegend), and then spun, blocked, and stained with an antibody mixture for 20 minutes (Supplemental Table 5), washed with FACS buffer 3 times, and centrifuged at 350*g* for 5 minutes. Cells were resuspended in 4% paraformaldehyde (PFA) for 10 minutes at room temperature, washed with FACS buffer 3 times, and marker expression was detected using the BD LSRFortessa X-20 Cell Analyzer (BD Biosciences) or the Attune Xenith Multispectral (Thermo Fisher Scientific).

### Co-IP.

To verify the potential binding of PDPN (expressed on GBM cells) and CLEC5A (expressed on TAMs), co-IP assays were conducted using the Pierce Co-Immunoprecipitation Kit (26149, Pierce, Thermo Fisher Scientific) according to the manufacturer’s protocol. OE-CLEC5A THP-1 cells alone, NU02068 neurospheres alone, THP-1 cells cocultured with NU02068 neurospheres and OE-CLEC5A THP-1 cells cocultured with NU02068 neurospheres were prepared for the co-IP assay. Furthermore, PDPN function was blocked in NU02068 neurospheres using InVivoMAb anti-PDPN (gp38) (BE0236, Bio X Cell), and these cells were cocultured with OE-CLEC5A THP-1 cells. Then, cell lysates were incubated with primary antibodies and normal IgG antibody (Supplemental Table 4). Bound proteins (PDPN, CLEC5A, and Flag) were detected by immunoblotting.

### PLA.

To assess the in-cell proximity of endogenous PDPN and CLEC5A with single-molecule sensitivity, we performed a PLA (DUO92101, MilliporeSigma) according to the manufacturer’s protocol. The PLA yields discrete fluorescent puncta only when 2 primary antibodies (different host species) bind targets located within 40 nm, providing high-specificity evidence of interaction in situ. Tumor-TAM (both THP-1 cells and primary monocytes) coculture systems were established under normoxia (21% O_2_) or hypoxia (1% O_2_). For loss-of-function studies, PDPN was knocked down in neurospheres and CLEC5A in THP-1–derived macrophages using shRNA constructs. Cells were incubated with PDPN and CLEC5A primary antibodies (Supplemental Table 4). Images were obtained with a ZEISS microscope. PLA signals were quantified as the number of puncta per defined image area (0.05 mm^2^), with identical thresholds applied across conditions.

### BLI.

The following recombinant proteins were used: human PDPN protein, Fc tag, multiangle light scattering-verified (MALS-verified), PON-H5254 (100 μg; ACROBiosystems); and human CLEC2/CLEC1B protein, His tag, MALS-verified, CL2-H5247 (100 μg; ACROBiosystems). Human CLEC5A/MDL-1 protein (HEK293, His), HY-P704543 (MedChem Express) were used to obtain real-time association (Kon) and dissociation (Koff) kinetics and the equilibrium affinity (*K_D_* = Koff/Kon) from globally fitted, multiconcentration sensorgrams. To validate PDPN-CLEC5A binding, we conducted a BLI assay using BLItz system (Keck Biophysics Facility, Northwestern University) according to the manufacturer’s protocol, with low-density ligand loading, reference subtraction, and replicate runs to limit mass transport and affinity artifacts. As a positive control, PDPN-CLEC2 binding was first validated under identical conditions, PDPN-CLEC5A binding was then assessed.

### Bone marrow collection, irradiation, and BMT.

To investigate the effect of CLEC5A KO in TAMs with regard to TAM polarization, the immunosuppressive TME, and tumor progression, we performed BMT using *Clec5a^–/–^*–transgenic mice (13791, Taconic Biosciences), with WT mice as the donors. Bone marrow collection and transplantation were conducted as previously described (26). All donor mice were euthanized by CO_2_ followed by secondary cervical dislocation, and femurs were collected. Bone marrow cells were washed out with PBS using a syringe with a 27 G needle, collected, and then placed through a sterile strainer. Cells were counted and resuspended to 2 × 10^7^ cells/mL in HBSS. Recipient mice were subjected to whole-body radiation exposure while anesthetized and inside a custom-designed head shield that protected the brain from radiation with 4 cm of lead. Mice received a radiation dose of 1.05 Gy/min for a duration of 10.5 minutes. Subsequently, mice were returned to their cages and administered i.v. bone marrow injections retro-orbitally (100 μL, containing 2 × 10^6^ cells) the following day. During the period required for immune reconstitution (4 weeks), mice were given TMS antibiotics (sulfamethoxazole and trimethoprim at 100 mg/L and 20 mg/L, respectively) in their drinking water.

### RCAS/tv-a mouse model and MRI scan.

The RCAS/tv-a system was used for somatic, cell-type–restricted gene delivery in glioma models. RCAS retroviral vectors selectively infect cells expressing the tv-a receptor, enabling targeted gene transfer in Nestin-tv-a–transgenic mice (26, 53). Nestin-tv-a *p53^fl/fl^* *Pten^fl/fl^* background (NpP) mice were donated by the Oren Becher laboratory (Mount Sinai Hospital, New York, New York, USA). Mice of both sexes (approximately equal distribution) and aged 8 weeks or older were used for all in vivo experiments. DF-1 chicken fibroblasts were transfected with either RCAS-Cre (RCAS-Cre-shPDPN) or RCAS-PDGFB-RFP constructs using Lipofectamine 3000 (L3000015, Invitrogen, Thermo Fisher Scientific) according to the manufacturer’s protocol (26). DF-1 cells were mixed at a 1:1 ratio prior to intracranial injection. RCAS-PDGFB was codelivered with RCAS-Cre to induce Pten and p53 loss selectively in RCAS-infected tv-a^+^progenitor cells, thereby initiating gliomagenesis in the intended lineage and anatomical location. A straight incision revealed skull features, allowing a syringe (1702, Hamilton Gastight) to be stereotactically positioned at 1–1.5 mm anterior and 1 mm lateral to bregma. A total of 5.0 × 10^5^ DF-1 cells in 1–3 μL culture medium were slowly injected at 500 nL/min using the microsyringe pump controller. Following injection, the needle rested for 1 minute and was then slowly removed. The skull hole was filled using sterile bone wax (Z046, SMI), and the incision was closed using absorbable sutures (MV-Z397-V, Oasis). Mice were monitored visually twice a week, and MRI scans were used to ensure tumor presence 2–3 weeks following injection. Body weights of the mice were monitored every 3 days. RCAS-shPDPN-Cre plasmids (sh-mPDPN-1 5′-GCTGCATCTTTCTGGATAATA-3′; sh-mPDPN-2 5′-CAGACAACAGATAAGAAAGAT-3′) were constructed by GenScript. Subsequently, the mice were euthanized, and their brains were carefully extracted for flow cytometry or fixed in 4% PFA and embedded in paraffin for H&E and IHC analysis. MRIs were captured using a Bruker ClinScan 7T MRI system operated via a Siemens Syngo platform. Anesthesia was maintained throughout the scan session. T2-weighted imaging (T2WI) and 3D T2WI sequence images were captured to monitor the tumor size and tumor growth patterns and analyzed using 3D Slicer software. All MRI scans were conducted by the Center for Translational Imaging (CTI) at Northwestern University.

### Statistics.

All experiments were performed at least 3 times, and all quantitative data are presented as the mean ± SD. Spearman correlation coefficients were used to assess the similarity matrix analysis and the relationship between CLEC5A and PDPN expression. Statistical analysis was performed using SPSS 20 and GraphPad Prism 10 (GraphPad Software). Comparisons between 2 groups were analyzed using an unpaired, 2-tailed Student’s *t* test. Comparisons among 3 or more groups were analyzed using 1-way ANOVA followed by Tukey’s or Dunnett’s multiple-comparison test. For experiments involving 2 independent variables, statistical significance was determined using 2-way ANOVA followed by Šídák’s, Tukey’s, or Dunnett’s multiple-comparison correction as appropriate for the experimental design and comparison strategy. Survival curves were compared using the log-rank (Mantel-Cox) test. A *P* value of less than 0.05 was considered statistically significant.

### Study approval.

All mouse procedures were approved by the IACUC of Northwestern University. Animals were bred and raised in conventional housing according to USDA standards. Animal welfare was ensured in accordance with the Northwestern Center for Comparative Medicine’s Rodent Standard of Care Policy.

Patient samples of surgically resected gliomas, together with pathological diagnoses, were obtained from the Department of Neurosurgery, Tianjin Medical University General Hospital, China, between August 2011 and April 2017, and were also collected from the Nervous System Tumor Bank (NSTB) at Northwestern University. Written informed consent was secured from all donors or their legal guardian. This study was conducted in accordance with the Helsinki Declaration and was approved by the ethics committees of Tianjin Medical University General Hospital and Northwestern University, respectively. All samples were histologically diagnosed by a certified neuropathologists based on WHO criteria.

### Data availability.

We downloaded the scRNA-seq GSE131928 dataset from the Gene Expression Omnibus (GEO) database (https://www.ncbi.nlm.nih.gov/geo), which contains data on 16,201 individual cells from 9 patients with IDH-WT GBM. We also expanded scRNA-seq analyses using a substantially larger and more deeply annotated public GBM scRNA-seq dataset available through the CZ Cell x Gene portal (https://cellxgene.cziscience.com/e/c888b684-6c51-431f-972a-6c963044cef0.cxg/) (25), which contains more than 338,000 cells and provides a comprehensive depiction of cellular composition through a harmonized, hierarchical cell annotation scheme. RNA-seq data, RNA microarray data, and corresponding clinical information (including WHO grades, histological types, IDH mutation status, and 1p/19q codeletion status) in The Cancer Genome Atlas (TCGA) and the CGGA were obtained from the University of California Santa Cruz (UCSC) databases website (https://xenabrowser.net/datapages/) and CGGA website (http://www.cgga.org.cn/), respectively. A total of 270 GBM RNA samples with corresponding anatomic structure annotation were obtained from the Ivy GAP (http://glioblastoma.alleninstitute.org/) to characterize *CLEC5A*, *PDPN*, and other transcripts within anatomic structures. Quantitative data underlying all graphs and statistical analyses are provided in the Supporting Data Values file. Custom analysis scripts and additional data supporting the findings of this study are available from the corresponding author upon reasonable request. Human participant data were deidentified prior to analysis and are shared in accordance with IRB policies and applicable privacy regulations.

## Author contributions

JL, XW, LT, BF, DF, RS, XY, and DJB designed the study. JL, XW, LT, BF, and LKS performed the bioinformatics analysis. JL, XW, BF, LKS, SMM, NGL, PB, and HN conducted the in vivo experiments. JL, XW, LT, and XY prepared the glioma tissue microarray. JL, XW, BF, LKS, SMM, TG, PB, and ERY performed the in vitro experiments. JL, XW, BF, LKS, XY, and DJB performed data analysis and interpretation. JL wrote the original draft of the manuscript. DJB and XY supervised the project and provided funding support. All authors were involved in the writing and editing of the manuscript and read and approved the final version of the manuscript.

## Conflict of interest

The authors have declared that no conflict of interest exists.

## Funding support

This work is the result of NIH funding, in whole or in part, and is subject to the NIH Public Access Policy. Through acceptance of this federal funding, the NIH has been given a right to make the work publicly available in PubMed Central.

- National Cancer Institute (NCI), NIH (R01CA214928 Identification and Targeting of Mechanisms Specific to Glioma stem cells in Glioblastoma; R01CA247905 Modeling the Glioblastoma Microenvironment to Uncover Progression Mechanisms and Therapeutic Targets; R01CA295560, Disrupting the Perinecrotic Engine of Immunosuppression in Glioblastoma; and P50CA221747 SPORE for Translational Approaches to Brain Cancer).
- Department of Defense (HT94252510613, A Mouse Model to Investigate Differential Response to Therapy and Mechanisms of Recurrence in Glioblastoma).
- National Natural Science Foundation of China (award nos. 82373151, 82103396, and 81872063).

## Supplementary Material

Supplemental data

Unedited blot and gel images

Supporting data values

## Version 1 06/02/2026

In-Press Preview

## Version 2 07/15/2026

Electronic publication
