# Supplementary material for: The perinecrotic niche of glioblastoma drives tumor-associated macrophage polarization and immunosuppression via podoplanin-mediated CLEC5A activation
Source: J Clin Invest. 2026 Jun 2;136(14):e199228. doi: 10.1172/JCI199228 (PMC13367977; doi:10.1172/JCI199228)
Supplement: Supplemental data [file jci-136-199228-s008.pdf]

Supplementary Figure 1

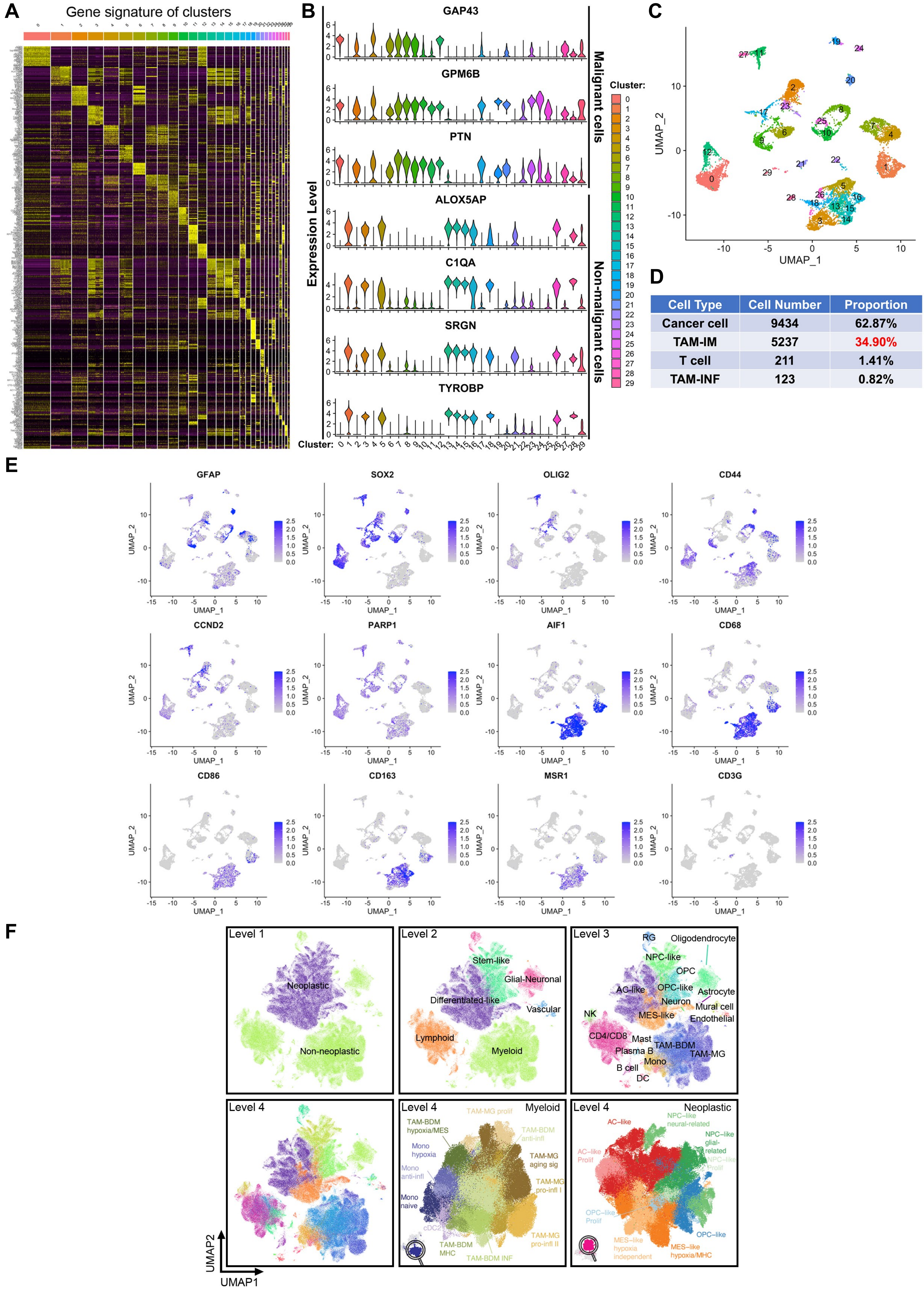

Cell Type	Cell Number	Proportion
Cancer cell	9434	62.87%
TAM-IM	5237	34.90%
T cell	211	1.41%
TAM-INF	123	0.82%

Supplementary Figure 2

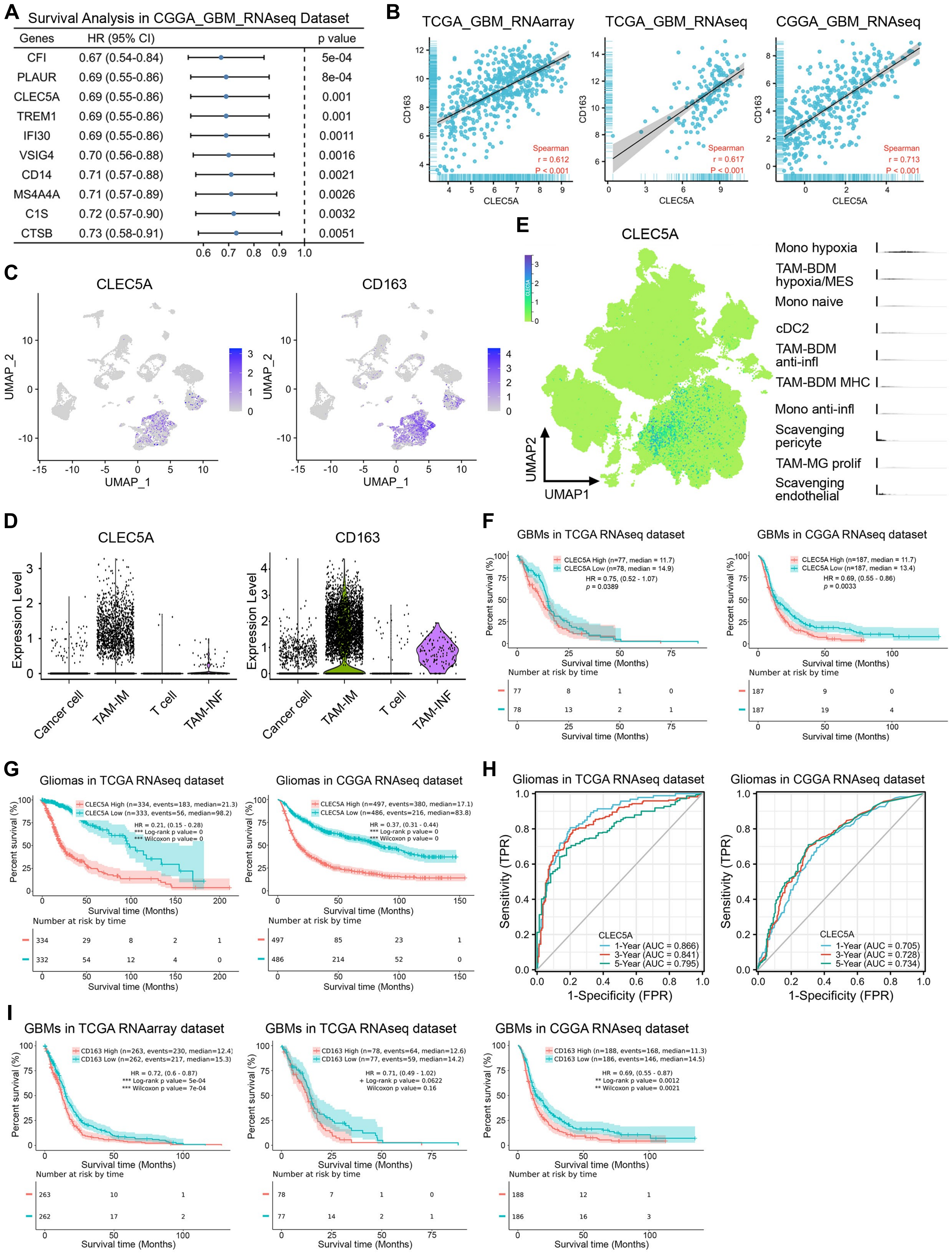

Supplementary Figure 3

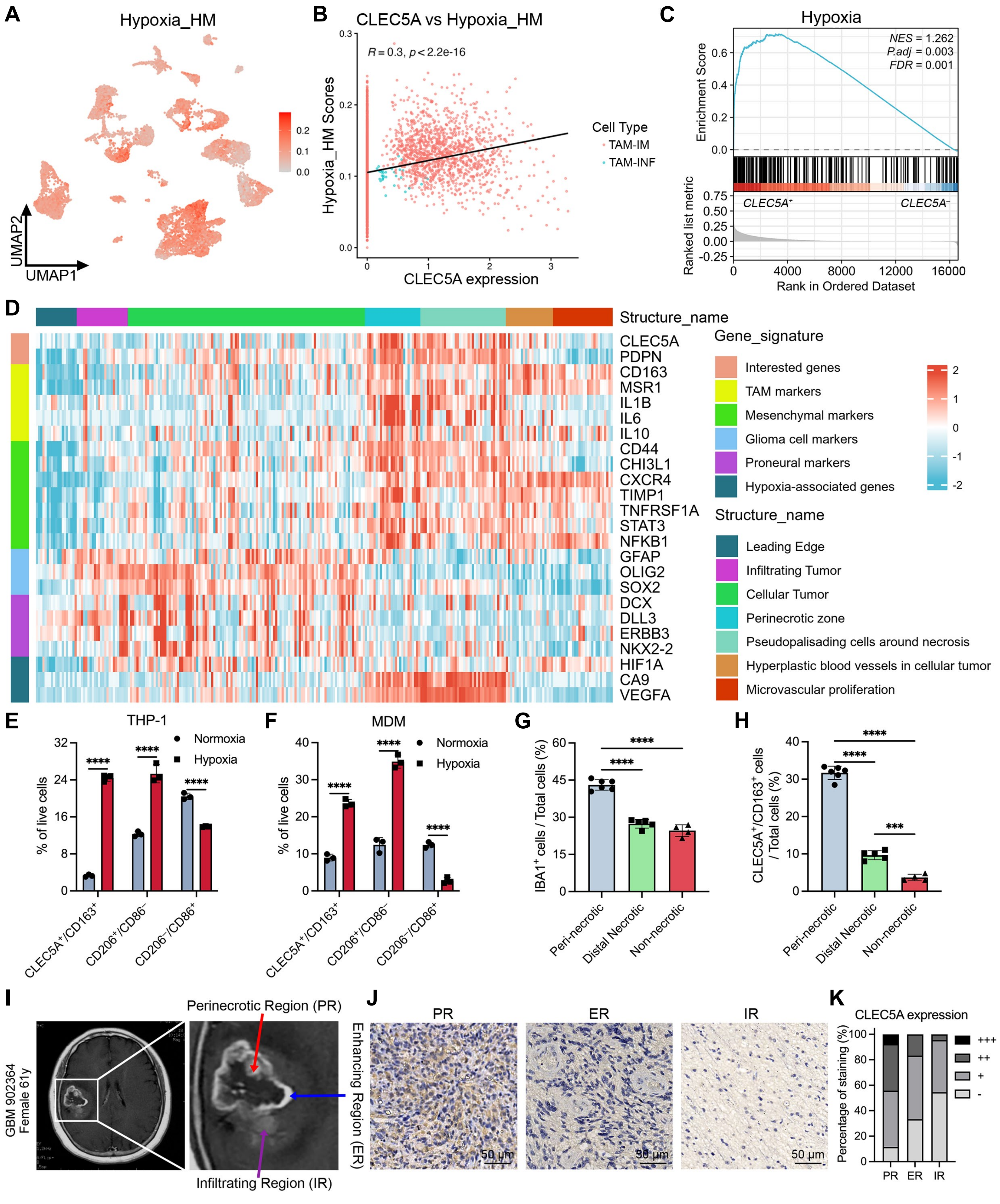

Supplementary Figure 4

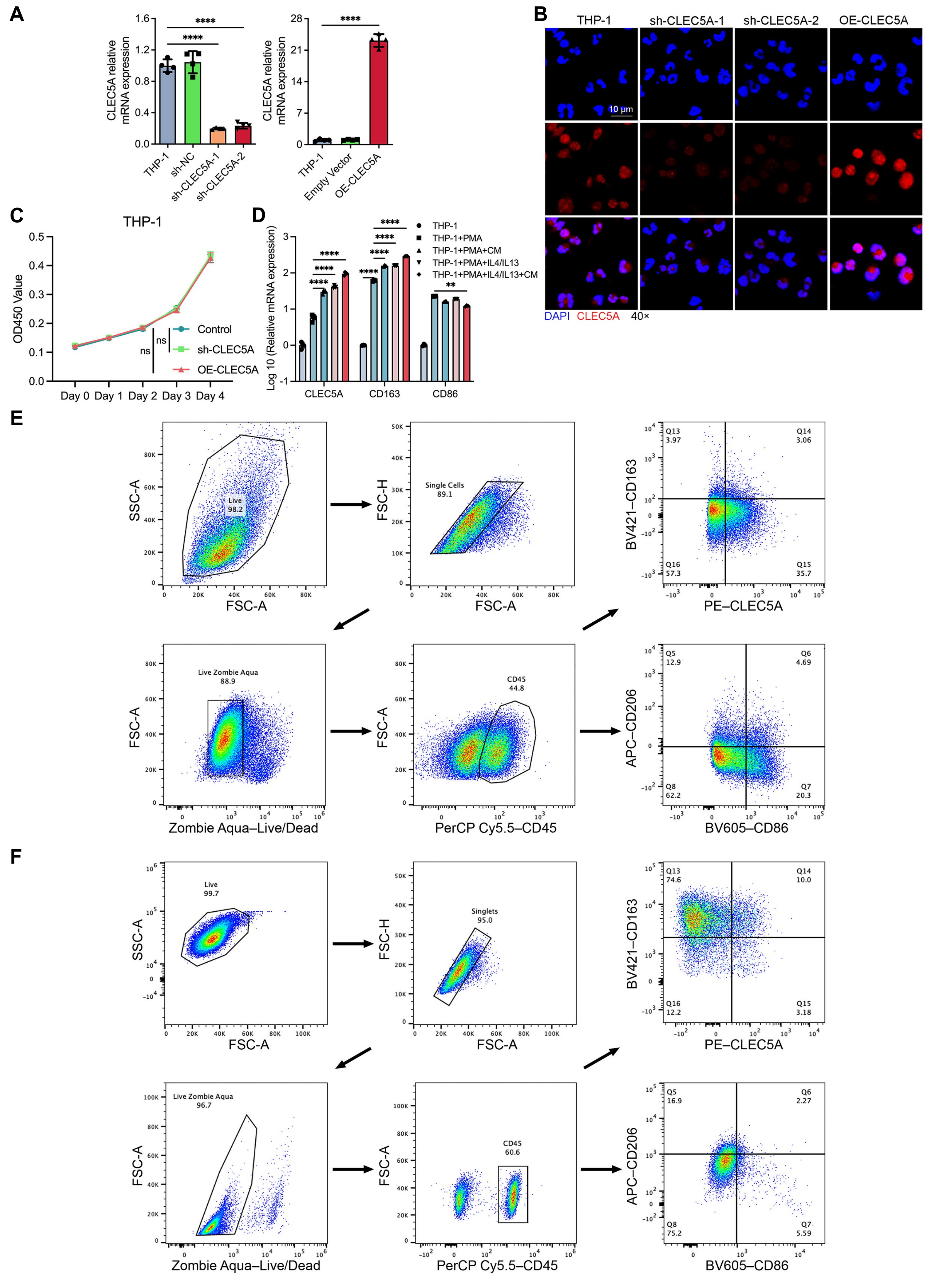

Supplementary Figure 5

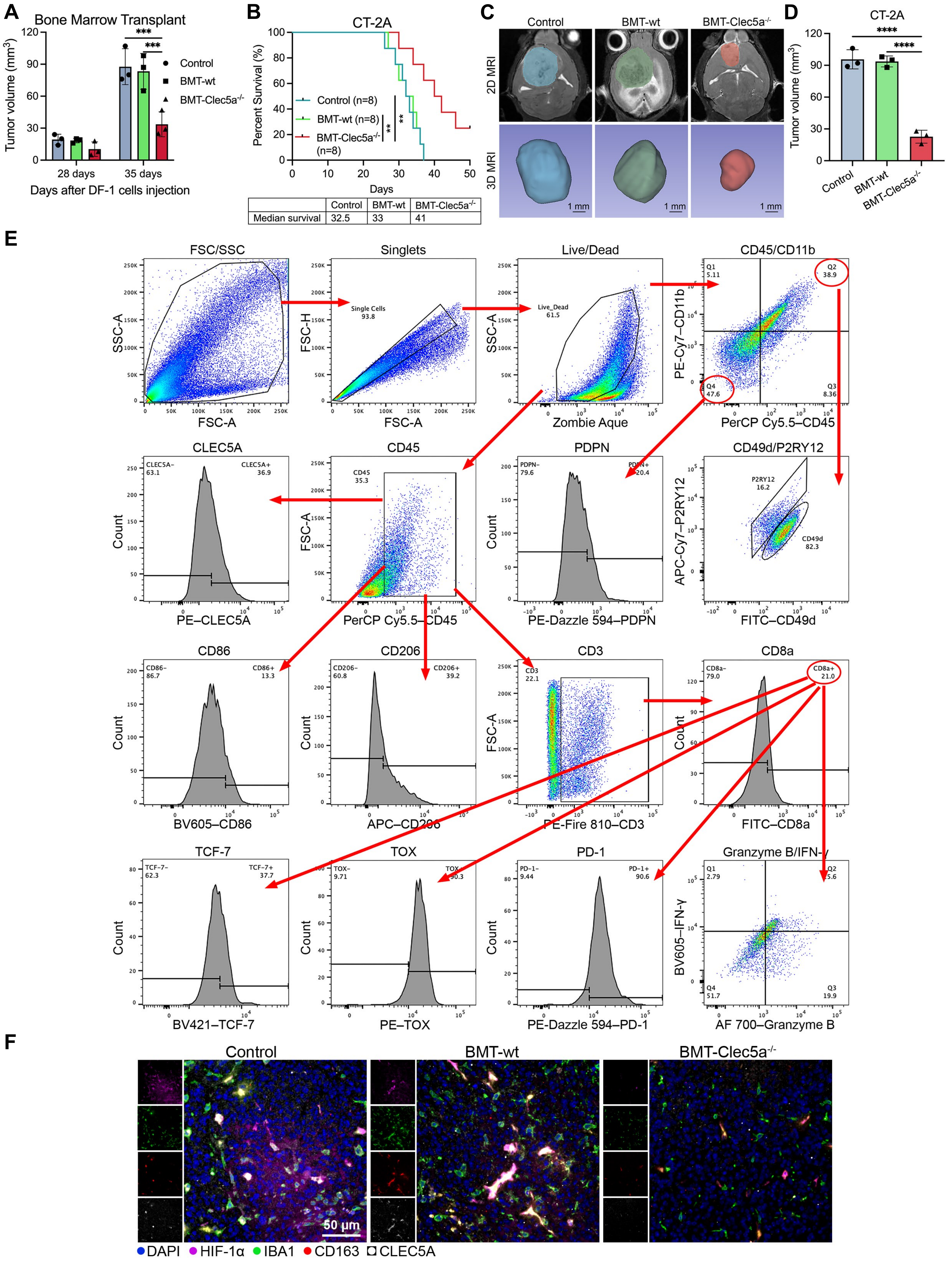

	Control	BMT-wt	BMT-Clec5a-/-
Median survival	32.5	33	41

Supplementary Figure 6

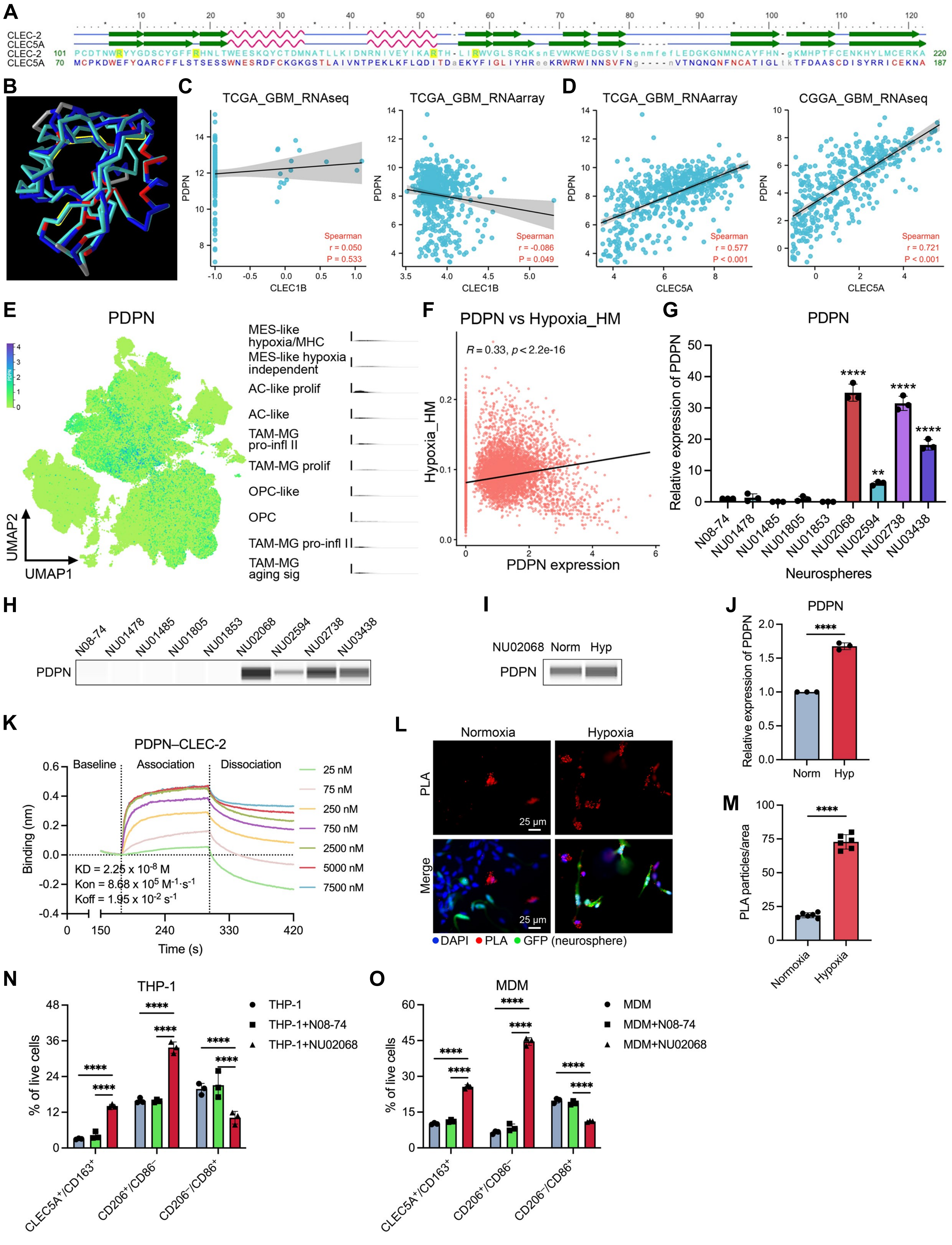

Supplementary Figure 7

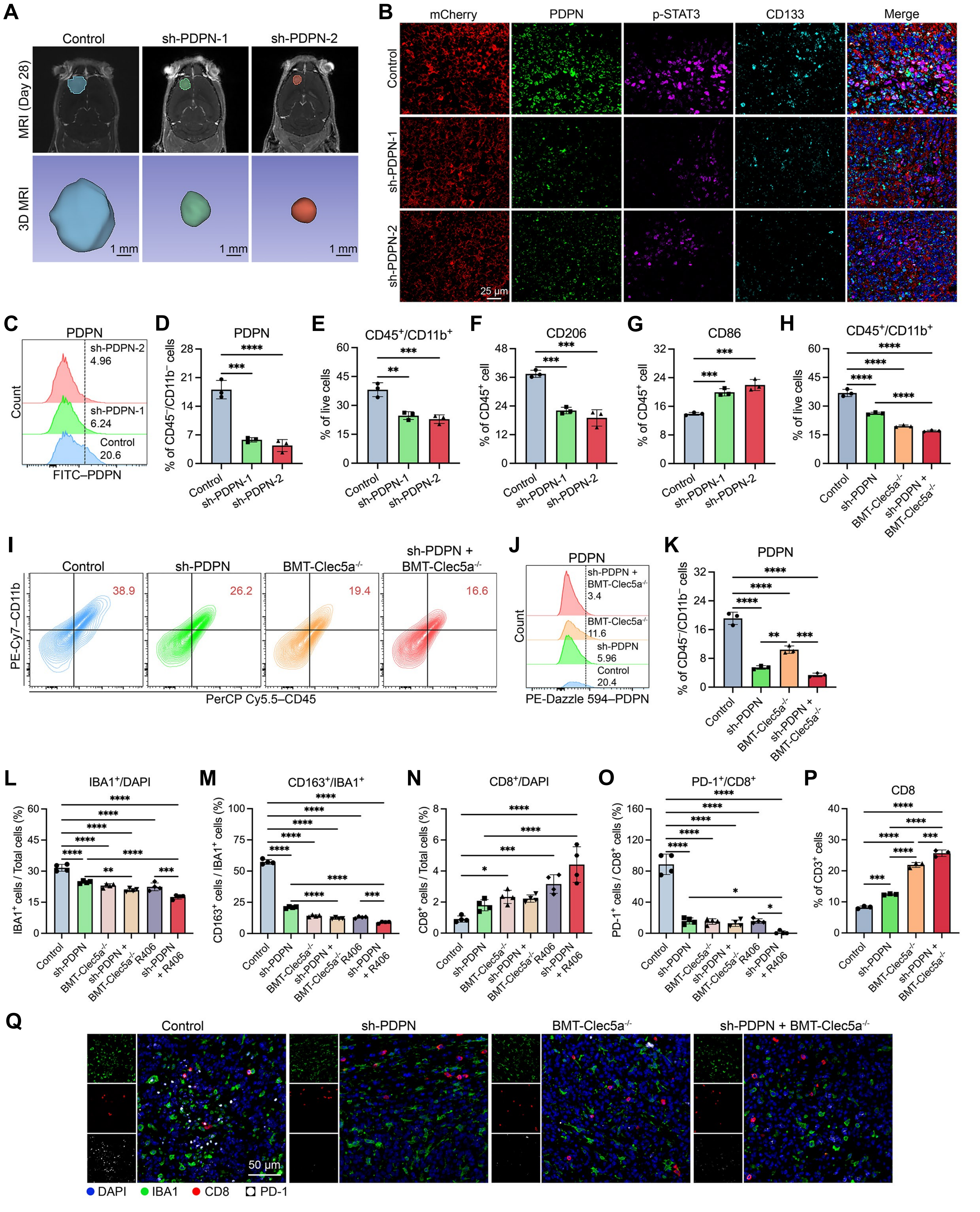

Supplementary Figure 8

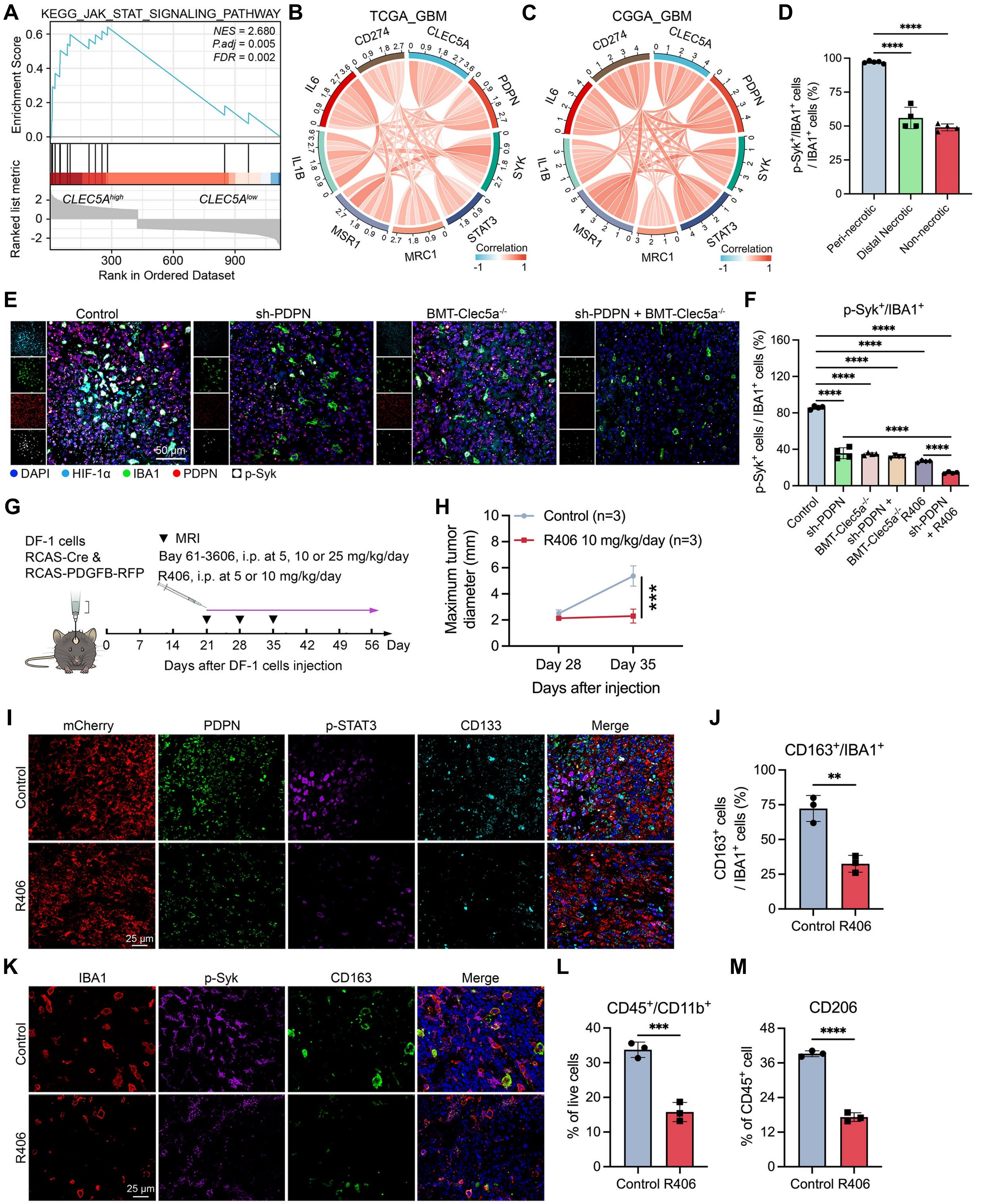

Supplementary Figure 9

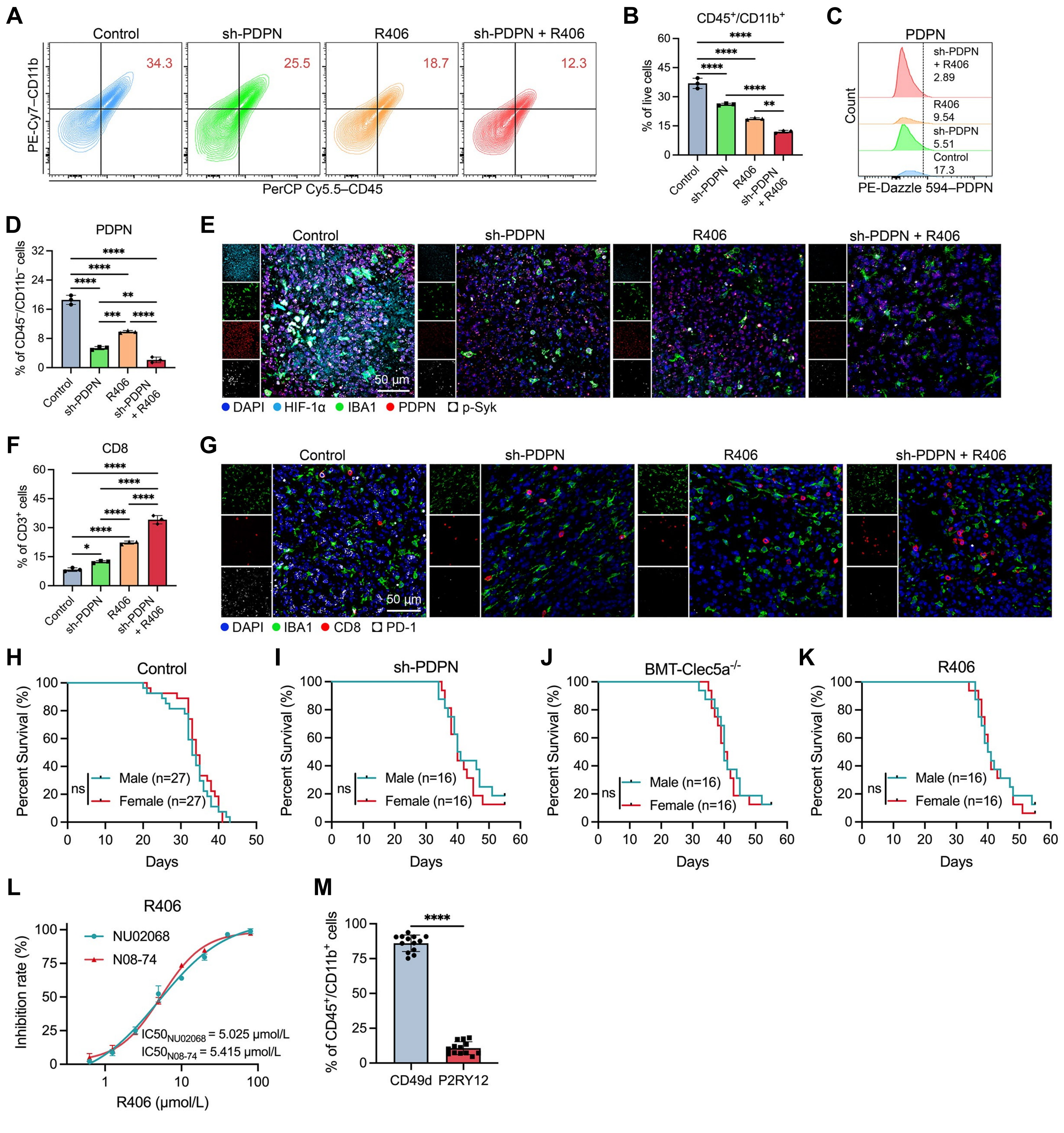

Supplementary Figure Legends

Supplementary Figure 1. scRNA-seq analysis

A. Heatmap clustering of GBM scRNA-seq data, using top 20 genes expressed for each cluster. **B.** Violin plot showing malignant cell markers (*GAP43, GPM6B* and *PTN*) and non-malignant cell markers (*ALOX5AP, C1QA, SRGN* and *TYROBP*) in specific clusters. **C.** scRNA-seq *Seurat* and UMAP analysis clustered samples into 30 distinct gene expression profiles. **D.** Table displaying scRNA-seq cellular subset numbers and proportions. **E.** Gene expression markers identify cell type signatures. **F.** UMAP projections annotated at increasing levels of resolution (Levels 1-4), illustrating separation of neoplastic and non-neoplastic compartments (Level 1), major lineage classes (Level 2), canonical immune and stromal subsets (Level 3), and refined cellular states including TAM subtypes and neoplastic programs (Level 4).

Supplementary Figure 2. CLEC5A is highly correlated with CD163 and survival curve analysis based on CLEC5A expression in gliomas

A. Forest plot validates the top 10 common genes closely associated with survival of GBMs, with *CLEC5A* ranking third in CGGA RNA-seq dataset. **B.** Spearman correlation coefficient shows correlation between *CD163* and *CLEC5A* in IDH-wildtype GBM in TCGA RNA microarray, TCGA RNA-seq and CGGA RNA-seq datasets. **C-D.** Feature plot (C) and violin plot (D) showing *CLEC5A* and *CD163* expression in specific cellular subsets within scRNA-seq data. **E.** Feature plot and distribution analysis of *CLEC5A* expression across cell populations. *CLEC5A* is enriched in hypoxia-associated myeloid states, including Mono hypoxia and TAM-BDM hypoxia/MES subsets. **F.** Kapan-Meier overall survival for GBM patients with high vs low *CLEC5A* expression in TCGA RNA-seq and CGGA RNA-seq datasets. **G.** Kapan-Meier survival curves for glioma patients with high vs low *CLEC5A* expression in TCGA RNA-seq and CGGA RNA-seq datasets. **H.** Time-dependent ROC curves display the predictive capacity of *CLEC5A* expression on 1-year, 3-year and 5-year glioma patient survival in TCGA and CGGA databases. **I.** Kapan-Meier overall survival for GBM patients with high vs low *CD163* expression in TCGA RNA microarray, TCGA RNA-seq and CGGA RNA-seq datasets. (**$p < 0.01$, ***$p < 0.001$)

Supplementary Figure 3. CLEC5A is enriched in hypoxic peri-necrotic niche of GBM

A. Feature plot of hypoxia gene set scores (MSigDB Hallmark Hypoxia; Hypoxia_HM) computed using the “Ucell” algorithm and projected onto the UMAP. **B.** Correlation analysis between

CLEC5A expression and hypoxia gene set scores in TAM-IM and TAM-INF subsets. **C**. GSEA analysis shows that *CLEC5A*$^{+}$ TAMs-IM are highly enriched in the "hypoxia" gene signature. **D.** Ivy GAP data demonstrating the micro-regional expression of *CLEC5A*, *PDPN*, TAM-IM markers (*CD163, MSR1, IL6, IL10*), mesenchymal markers (*CD44, CHI3L1, CXCR4, TIMP1, TNFRSF1A, STAT3, NFKB1*), cancer cells markers (*GFAP, OLIG2, SOX2*), and proneural markers (*OLIG2, SOX2, DCX, DLL3, ERBB3, NKX2-2*) in 270 samples from 44 GBMs with 7 anatomical subregions, including the leading edge (LE), infiltrating tumor (IT), cellular tumor (CT), microvascular proliferation (MVP), hyperplastic blood vessels (HBV), peri-necrotic zone (PZ), and pseudopalisading cells (PC). **E-F.** Quantification of CLEC5A^{+}/CD163^{+}, CD206^{+}/CD86^{-}, and CD206^{-}/CD86^{+} THP-1 populations (E) and MDM populations (F) under normoxia versus hypoxia (two-way ANOVA, Šídák's multiple-comparisons test). **G-H.** Quantification of IBA1^{+} cells as a percentage of total cells (G), and CLEC5A^{+}/CD163^{+} cells as a percentage of total cells (H) across peri-necrotic, distal necrotic, and non-necrotic regions (one-way ANOVA, Tukey's multiple-comparisons test). **I.** MRI imaging was used to identify tumor subregions: the peri-necrotic region (PR) adjacent to the necrotic core, the enhancing region (ER) surrounding the contrast-enhancing tumor, and the infiltrating region (IR) representing the non-enhancing infiltrative tumor. **J.** TMA representative images displaying CLEC5A in multiple regions from one GBM patient sample (Scale bar, 50 μm). **K.** Total CLEC5A IHC score in three multiregional of GBM samples (a total score of 0-1 as negative (-), 2-3 as weakly positive (+), 4-5 as moderately positive (++) and 6-7 as strongly positive (+++)). (****p* < 0.001, *****p* < 0.0001)

Supplementary Figure 4. Validation of CLEC5A knockdown and overexpression efficiency in THP-1 cells and flow cytometry gating strategy

A. qPCR showing lentiviral transfection efficiency for knockdown (sh-CLEC5A-1, sh-CLEC5A-2) and overexpression (OE-CLEC5A) vectors along with the resultant *CLEC5A* mRNA expression in THP-1 cells (one-way ANOVA, Dunnett's multiple-comparisons test). **B.** IF staining displaying lentiviral transfection efficiency for knockdown (sh-CLEC5A-1, sh-CLEC5A-2) and overexpression (OE-CLEC5A) vectors (Scale bar, 10 μm). **C.** Cell proliferation of THP-1 cells with control, CLEC5A knockdown (sh-CLEC5A), or CLEC5A overexpression (OE-CLEC5A) measured by CCK-8 assay over 4 days. OD450 values are shown at indicated time points. **D.** *CLEC5A, CD163* and *CD86* mRNA expression levels in THP-1 cells treated with PMA, IL-4, IL-13 and/or glioma CM analyzed using qPCR (two-way ANOVA, Tukey's multiple-comparisons

test). **E-F.** Flow cytometry gating strategy used for TAM phenotyping in THP-1 cells (E) and primary human PBMC-derived MDMs (F). Sequential gating included FSC/SSC selection, singlet discrimination, live/dead gating, and $CD45^+$ cell identification prior to analysis of CLEC5A and TAM polarization markers. ($^{ns}p > 0.05$, $^*p < 0.05$, $^{**}p < 0.01$, $^{***}p < 0.001$, $^{****}p < 0.0001$)

Supplementary Figure 5. CLEC5A deletion in TAMs suppresses TME immunosuppression

A. Histogram showing tumor volume of tumor-bearing mice in control, BMT-wt and BMT-Clec5a-/- groups at 28 and 35 days following DF-1 cells injection (two-way ANOVA, Tukey's multiple-comparisons test). **B.** Kaplan-Meier survival analysis of mice bearing orthotopic CT-2A tumors in Control, BMT-wt, and BMT-Clec5a-/- groups (n = 8 per group). Median survival is indicated. **C.** Representative 2D MRI images (top) and corresponding 3D tumor reconstructions (bottom) from each group at 32 days post-injection. Tumor boundaries are outlined (Scale bars, 1 mm). **D.** Quantification of tumor volume based on MRI-derived 3D reconstruction across groups (one-way ANOVA, Tukey's multiple-comparisons test). **E.** Flow cytometry gating strategy for myeloid and T cell phenotyping in mouse brain tumors. Sequential gating strategy used for flow cytometric analysis of dissociated mouse brain tumor tissues. Cells were first gated by FSC/SSC to exclude debris, followed by singlet discrimination and live/dead exclusion. $CD45^+/CD11b^+$ myeloid cells were identified and further subdivided into $CD49d^+$ bone marrow-derived macrophages and $P2RY12^+$ microglia. $PDPN^+$ cells were assessed within $CD45^-/CD11b^-$ cells. CLEC5A and TAM polarization markers CD206 and CD86 were analyzed within $CD45^+$ cells. Within the $CD45^+/CD3^+$ lymphoid compartment, $CD8^+$ T cells were further characterized for activated cytotoxic markers Granzyme B and IFN-γ, and exhaustion markers, including PD-1, TOX (terminal exhausted) and TCF-7 (precursor). Representative plots are shown for each gating step, with percentages indicating gated populations. **F.** IF staining of HIF-1α, IBA1, CD163 and CLEC5A in control, BMT-wt and BMT-$Clec5a^{-/-}$ mice gliomas (Scale bar, 50 μm). ($^*p < 0.05$, $^{**}p < 0.01$, $^{***}p < 0.001$, $^{****}p < 0.0001$)

Supplementary Figure 6. PDPN is associated with CLEC5A and triggers TAM polarization

A-B. Comparison of domains, amino acid sequence and 3-dimensional protein structures of CLEC5A and CLEC-2. **C-D.** Spearman correlation coefficient analysis for *PDPN* expression and *CLEC1B* (*CLEC-2*) (C) as well as *CLEC5A* (D) in TCGA and CGGA GBM datasets. **E.** Feature plot and distribution analysis of PDPN expression across cell populations. PDPN is enriched in

neoplastic cell states, particularly MES-like hypoxia/MHC and MES-like hypoxia-independent programs. **F.** Correlation analysis between PDPN expression and hypoxia gene set scores in cancer cells. **G-H.** The protein expression level of PDPN in different human primary neurospheres (one-way ANOVA, Dunnett's multiple-comparisons test). **I-J.** The protein expression level of PDPN in normoxic and hypoxic conditions in NU02068 neurospheres (unpaired two-tailed Student's t tests). **K.** BLI analysis demonstrates direct cell-free binding of PDPN to CLEC-2, with kinetic binding (Koff/Kon) and equilibrium affinity (KD) derived from global kinetic fitting. **L-M.** Representative PLA images and quantification of PLA puncta in co-cultures of human PBMC-derived MDMs and patient-derived neurospheres under normoxia or hypoxia (Scale bar, 25 μm) (unpaired two-tailed Student's t tests). **N-O.** Quantification of THP-1 cells (N) and MDMs (O) co-cultured with high-PDPN expression neurospheres (NU02068) or low-PDPN expression neurospheres (N08-74) (two-way ANOVA, Tukey's multiple-comparisons test). (*$p < 0.05$, **$p < 0.01$, ***$p < 0.001$, ****$p < 0.0001$)

Supplementary Figure 7. Suppressing PDPN reduces tumor progression *in vivo*

A. Representative 2D MRI images (top) and corresponding 3D tumor reconstructions (bottom) of RCAS/tv-a tumor-bearing mice at day 28 following intracranial injection of DF-1 cells carrying RCAS control or PDPN knockdown constructs (sh-PDPN-1 and sh-PDPN-2). Tumor boundaries are outlined (Scale bars, 1 mm). **B.** IF staining of mCherry (glioma cells), PDPN, p-STAT3 and CD133 in control and sh-PDPNs mice gliomas (Scale bar, 25 μm). **C-D.** Representative flow cytometry images and quantification showing expression of PDPN in control and sh-PDPNs mouse gliomas (one-way ANOVA, Dunnett's multiple-comparisons test). **E-G.** Quantification of $CD45^+/CD11b^+$ myeloid cells as a percentage of live cells (E), CD206 (F), and CD86 (G) expression within $CD45^+$ myeloid cells (one-way ANOVA, Dunnett's multiple-comparisons test). **H-I.** Representative flow cytometry plots and quantification of $CD45^+/CD11b^+$ myeloid cells as a percentage of live cells across Control, sh-PDPN, BMT-$Clec5a^{-/-}$, and sh-PDPN + BMT-$Clec5a^{-/-}$ groups (one-way ANOVA, Tukey's multiple-comparisons test). **J-K.** Representative flow cytometry images and quantification showing expression of PDPN among $CD45^–/CD11b^–$ cells across indicated groups (one-way ANOVA, Tukey's multiple-comparisons test). **L-O.** Quantification of $IBA1^+$ cells as a percentage of total (DAPI) cells (L), $CD163^+$ cells as a percentage of $IBA1^+$ myeloid cells (M). $CD8^+$ cells as a percentage of total cells (N), and $PD\text{-}1^+$ cells as a percentage of $CD8^+$ T cells (O) in IF images across indicated groups (Control, sh-

PDPN, BMT-Clec5a$^{-/-}$, sh-PDPN + BMT-Clec5a$^{-/-}$, R406, and sh-PDPN + R406) (one-way ANOVA, Tukey's multiple-comparisons test). **P.** Quantification of CD8+ cells in CD3+ T cells across groups (one-way ANOVA, Tukey's multiple-comparisons test). **Q.** Representative multiplex IF images of mouse brain tumors showing IBA1, CD8 and PD-1 in control, sh-PDPN, BMT-Clec5a$^{-/-}$ and shPDPN + BMT-Clec5a$^{-/-}$ groups. (*$p < 0.05$, **$p < 0.01$, ***$p < 0.001$, ****$p < 0.0001$)

Supplementary Figure 8. CLEC5A promotes TAM-IM polarization and immunosuppressive TME through Syk-JAK-STAT3

A. GSEA showing *CLEC5A*-related genes are highly enriched in JAK-STAT signaling pathway using TCGA dataset (NES = 2.680, *p*.adj = 0.005). **B-C.** Chord diagram showing relationship between *CLEC5A* and *PDPN*, Syk signaling molecules (*Syk, STAT3*), as well as immunosuppressive-related genes (*MRC1, MSR1 and CD274*) using TCGA and CGGA GBM databases. **D.** Histogram depicting the proportion of p-Syk^{+}/IBA1^{+} cells relative to IBA1^{+} cells across three distinct areas: peri-necrotic areas, distal necrotic zones in necrotic tumors, and in non-necrotic gliomas by IF staining shown in Fig. 6B (one-way ANOVA, Dunnett's multiple-comparisons test). **E.** Representative images showing PDPN, p-Syk, IBA1, and HIF-1α expression across the indicated groups (Scale bar, 50 μm). **F.** Quantification of p-Syk^{+} cells as a percentage of IBA1^{+} myeloid cells in IF images across indicated groups (Control, sh-PDPN, BMT-Clec5a$^{-/-}$, sh-PDPN + BMT-Clec5a$^{-/-}$, R406, and sh-PDPN + R406) (one-way ANOVA, Tukey's multiple-comparisons test). **G.** Schematic diagram of *in vivo* experimental design using the RCAS/tv-a mouse model to evaluate the therapeutic effects of Bay (5, 10, or 25 mg/kg/day) and R406 (5, or 10 mg/kg/day). **H.** Histogram illustrating changes in maximum tumor diameter as observed in MRI images at days 28 and 35 following glioma induction. **I.** IF staining of mCherry (glioma cell), PDPN, p-STAT3 and CD133 in RCAS/tv-a mouse tissue sections (Scale bar, 25 μm). **J.** Histogram showing the proportion of CD163 expression in IBA1^{+} myeloid cells in control and R406 treated mice (unpaired two-tailed Student's t tests). **K.** IF staining of IBA1 (immune cell), p-Syk and CD163 in RCAS/tv-a mouse tissue sections (Scale bar, 25 μm). **L-M.** Histogram showing the proportion of CD45^{+}/CD11b^{+} cells among live cells (L) and CD206^{+} cells among the CD45^{+} population (M) in control and R406 treated mice through flow cytometry (unpaired two-tailed Student's t tests). (*$p < 0.05$, **$p < 0.01$, ***$p < 0.001$, ****$p < 0.0001$)

Supplementary Figure 9. PDPN knockdown combined with Syk inhibition suppresses tumor progression and reprograms the immunosuppressive TME

A-B. Representative flow cytometry plots and quantification of $CD45^{+}/CD11b^{+}$ myeloid cells in mouse brain tumors across indicated groups (Control, sh-PDPN, R406, and sh-PDPN + R406) (one-way ANOVA, Tukey's multiple-comparisons test). **C-D.** Representative flow cytometry plots and quantification of $PDPN^{+}$ cells among $CD45^{-}/CD11b^{-}$ in mouse brain tumors across indicated groups (one-way ANOVA, Tukey's multiple-comparisons test). **E.** Representative IF images showing PDPN, p-Syk, IBA1, and HIF-1α expression in tumor sections across groups (Scale bar, 50 μm). **F.** Quantification of CD8+ cells in CD3+ T cells across groups (one-way ANOVA, Tukey's multiple-comparisons test). **G.** Representative IF images showing IBA1, CD8 and PD-1 expression across treatment groups (Scale bar, 50 μm). **H-K.** Kaplan–Meier survival analyses stratified by sex (male vs female) in tumor-bearing mice under Control (H), PDPN knockdown (sh-PDPN) (I), Clec5a-deficient bone marrow transplantation (BMT-$Clec5a^{-/-}$) (J), and Syk inhibitor (R406) treatment (K) conditions. Group sizes are indicated in each panel. **L.** Dose-response curves of patient-derived GBM neurospheres treated with Syk inhibitor R406 through CCK-8 assay. **M.** Flow cytometry-based quantification of myeloid cell origin within $CD45^{+}CD11b^{+}$ tumor-infiltrating cells using CD49d and P2RY12 to distinguish monocyte-derived macrophages (mo-TAMs) and microglia. The majority of tumor-associated myeloid cells are $CD49d^{+}$ mo-TAMs (unpaired two-tailed Student's t tests). (*$p < 0.05$, **$p < 0.01$, ***$p < 0.001$, ****$p < 0.0001$)

Supplementary Tables

Supplementary Table 1

Survival analysis of common genes highly associated with *CD163*

Common genes	*p* value	HR	1-year AUC	2-year AUC
CD163			0.645	0.605
CLEC5A	1.16E-06	0.63 (0.52-0.76)	0.685	0.621
CTSB	0.0003	0.71 (0.59-0.86)	0.729	0.598
C1R	0.0005	0.72 (0.59-0.86)	0.646	0.592
FCGR2B	0.0006	0.72 (0.60-0.87)	0.627	0.64
C1S	0.0007	0.72 (0.60-0.87)	0.631	0.569
IFI30	0.0008	0.73 (0.60-0.88)	0.664	0.515
F13A1	0.0008	0.73 (0.60-0.88)	0.607	0.551
CFI	0.0018	0.74 (0.62-0.90)	0.644	0.627
ALOX5AP	0.0026	0.75 (0.62-0.91)	0.636	0.534
C5AR1	0.0028	0.75 (0.62-0.91)	0.672	0.568
FCGR2A	0.003	0.75 (0.62-0.91)	0.634	0.57
THBD	0.0043	0.76 (0.63-0.92)	0.659	0.597
CSTA	0.0058	0.77 (0.64-0.93)	0.626	0.629
TREM1	0.0063	0.77 (0.64-0.93)	0.656	0.618
SIGLEC7	0.0086	0.78 (0.65-0.94)	0.649	0.527
RNASE2	0.0142	0.79 (0.66-0.95)	0.635	0.567
LHFPL2	0.0154	0.79 (0.66-0.96)	0.611	0.489
TNFRSF1B	0.0175	0.80 (0.66-0.96)	0.635	0.543
CD14	0.0186	0.80 (0.66-0.96)	0.661	0.56
LAIR1	0.0248	0.81 (0.67-0.97)	0.655	0.551
HMOX1	0.0256	0.81 (0.67-0.97)	0.673	0.511
NCF4	0.0258	0.81 (0.67-0.97)	0.627	0.535
SLC11A1	0.0265	0.81 (0.67-0.98)	0.699	0.517
STAB1	0.0282	0.81 (0.67-0.98)	0.638	0.535
LILRB3	0.0294	0.81 (0.67-0.98)	0.713	0.58

Supplementary Table 2

Baseline characteristics table of glioma patients in TCGA database

Characteristic	Low expression of *CLEC5A*	High expression of *CLEC5A*	*p*
n	348	348	
WHO grade, n (%)			< 0.001
G2	163 (25.7%)	61 (9.6%)	
G3	135 (21.3%)	108 (17%)	
G4	5 (0.8%)	163 (25.7%)	
IDH status, n (%)			< 0.001
WT	22 (3.2%)	224 (32.7%)	
Mut	324 (47.2%)	116 (16.9%)	
1p/19q codeletion, n (%)			< 0.001
codel	155 (22.5%)	16 (2.3%)	
non-codel	193 (28%)	325 (47.2%)	
Primary therapy outcome, n (%)			< 0.001
PD	47 (10.2%)	65 (14.1%)	
SD	104 (22.5%)	43 (9.3%)	
PR	47 (10.2%)	17 (3.7%)	
CR	104 (22.5%)	35 (7.6%)	
Gender, n (%)			1.000
Female	149 (21.4%)	149 (21.4%)	
Male	199 (28.6%)	199 (28.6%)	
Age, n (%)			< 0.001
<=60	315 (45.3%)	238 (34.2%)	
>60	33 (4.7%)	110 (15.8%)	
Histological type, n (%)			< 0.001
Astrocytoma	98 (14.1%)	97 (13.9%)	
Glioblastoma	5 (0.7%)	163 (23.4%)	
Oligoastrocytoma	90 (12.9%)	44 (6.3%)	
Oligodendroglioma	155 (22.3%)	44 (6.3%)	
OS event, n (%)			< 0.001
Alive	284 (40.8%)	140 (20.1%)	
Dead	64 (9.2%)	208 (29.9%)	
Race, n (%)			0.297
Asian	5 (0.7%)	8 (1.2%)	
Black or African American	13 (1.9%)	20 (2.9%)	
White	325 (47.6%)	312 (45.7%)	
DSS event, n (%)			< 0.001
Alive	288 (42.7%)	143 (21.2%)	
Dead	56 (8.3%)	188 (27.9%)	
PFI event, n (%)			< 0.001
Alive	237 (34.1%)	113 (16.2%)	
Dead	111 (15.9%)	235 (33.8%)	
Age, mean ± SD	41.82 ± 13.32	51.53 ± 15.34	< 0.001

Supplementary Table 3

Cytokine antibody microarray

ID (pg/ml)	Control	Positive Control	sh-NC	sh-CLEC5A	Empty Vector	OE-CLEC5A
IL-1a	138	96.25	317.25	129	249.5	201.5
IL-1b	3675.13	4192.63	3594.88	1608.5	3730.25	4426.88
IL-2	932.38	2031	1016	651.88	663	1440.38
IL-4	113.5	145	128.75	0	124.25	162.25
IL-5	1166.75	2611.5	1065.13	947.38	942.63	1973.75
IL-6	633.63	1596.88	662.25	342.63	637.25	1234.25
IL-8	27474.75	53143.75	47229.75	23213.25	27729.25	75306.88
IL-10	4765.13	8638.75	4864.25	2534.75	4762.13	9917
IL-12p70	0	0	94.38	0	0	113.75
IL-13	1660	4104	1625.75	1018.25	1287.25	2024
GM-CSF	221	370.75	196.38	99	214.38	249.75
GRO	20023.88	22783.63	20330.38	10493.25	20484.25	19017
IFNg	1183.88	2119.38	1163.25	896.75	1026	1563.88
MCP-1	21029.75	21086.75	20884.75	15806.63	12634.88	19536
MIP-1a	142340.8	143224.9	141177	138468	130039.4	142316
MIP-1b	82885.38	82075.38	88608.5	73786.88	67980	86523.5
MMP-9	19130.5	11798	19436.63	12455.88	10023.38	14382.38
RANTES	155180.1	143262.4	161735.8	143325.8	147693	147578.8
TNFa	899.75	2053.25	888	560.63	891.63	1700.25
VEGF	12	0	431.25	107	215.25	89.25

Supplementary Table 4

Antibody table

Antibody	Source	Company	Cat. #	Application	Dilution
CAIX	mouse	Santa Cruz Biotech	sc-365900	IF	1:100
CD133	mouse	DSHB (Iowa)	HB#7 sup	IF	1:100
CD163	rabbit	Abcam	ab182422	WB	1:1000
CD163	rat	Abcam	ab289979	IF	1:200
CD163	mouse	Abcam	ab156769	IF	1:100
CD163	mouse	GeneTex	GTX54458	IF	1:250
CD163	rat	Invitrogen	14-1631-82	IF	1:200
CD206	rabbit	ABclonal	A11192	WB	1:1000
CD8 alpha	rabbit	Abcam	ab316778	IF	1:200
CLEC5A	rabbit	Abcam	ab203200	IHC	1:100
CLEC5A	rabbit	Abcam	ab184156	IF	1:100
				WB	1:1000
CLEC5A	rabbit	Abcam	ab314870	IF	1:100
CLEC5A	rabbit	Bioss	bs-2663R	PLA	1:200
FLAG DYKDDDDK	rabbit	CST	14793	co-IP	1:50
HIF-1α	mouse	CST	79233S	IF	1:100
IBA1	chicken	Aves Labs	IBA1-0020	IF	1:400
Jak2	rabbit	CST	3230S	WB	1:1000
mCherry	chicken	Aves Labs	mcherry-0100	IF	1:400
Normal IgG	rabbit	CST	2729S	co-IP	1:200
PD-1	mouse	Abcam	ab52587	IF	1:100
PD-1	rat	Abcam	ab309360	IF	1:100
PD-L1	rabbit	CST	13684S	WB	1:1000
PDPN	rabbit	CST	9047S	co-IP	1:200
PDPN	mouse	Santa Cruz Biotech	sc-59347	IF	1:100
				PLA	1:200
PDPN	rat	Invitrogen	MA5-16267	IF	1:200
PDPN	rat	Invitrogen	MA5-16270	IF	1:100
phospho-Jak2	rabbit	CST	3771S	WB	1:1000
phospho-STAT3	rabbit	CST	9145S	IF	1:100
				WB	1:1000
phospho-Syk	rabbit	CST	2710S	IF	1:100
				WB	1:1000
STAT3	mouse	CST	9139S	WB	1:1000
Syk	rabbit	CST	13198S	WB	1:1000
β-actin	mouse	ZSGB-Bio	TA-09	WB	1:2000

Supplementary Table 5

Flow cytometry antibody table

Antibody	Species	Fluorophore	Company	Cat. #	Dilution
CD11b	rat anti-human/mouse	PE-Cy7	BD Biosciences	552850	1:50
CD163	mouse anti-human	BV421	BioLegend	333612	1:20
CD206	mouse anti-human	APC	BioLegend	321110	1:20
CD206	rat anti-mouse	APC	BioLegend	141708	1:25
CD279 (PD-1)	rat anti-mouse	PE/Dazzle 594	BioLegend	135228	1:50
CD3	rat anti-mouse	PE/Fire 810	BioLegend	100277	1:20
CD45	rat anti-mouse	PerCP/Cyanine5.5	BioLegend	103132	1:20
CD45	mouse anti-human	PerCP/Cyanine5.5	BioLegend	368503	1:20
CD49d	rat anti-mouse	FITC	BioLegend	103606	1:50
CD8a	rat anti-mouse	AF488	BioLegend	100723	1:50
CD86	mouse anti-human	BV605	BioLegend	374214	1:20
CD86	rat anti-mouse	BV605	BioLegend	105125	1:25
CLEC5A	mouse anti-human	PE	BioLegend	371705	1:50
CLEC5A	human anti-mouse	PE	Miltenyi Biotec	130-126-308	1:50
Granzyme B	rat anti-human/mouse	AF700	BioLegend	396426	1:20
IFN-γ	rat anti-mouse	BV605	BioLegend	505839	1:25
P2RY12	rat anti-mouse	APC/Cyanine7	BioLegend	848024	1:40
PDPN	syrian hamster anti-mouse	FITC	BioLegend	127416	1:50
PDPN	rat anti-human/mouse	PE-Dazzle 594	BioLegend	337028	1:20
TCF-7	mouse anti-human/mouse	BV421	BD Biosciences	566692	1:20
TOX	human anti-human/mouse	PE	Miltenyi Biotec	130-120-716	1:50

Supplementary Table 6

qPCR primer sequences

Targets	Primer sequences
CLEC5A	Forward: 5'-GTGGCGTTGGATCAACAACT-3'
	Reverse: 5'-CTTCTCACAGATCCTGCGGT-3'
CD163	Forward: 5'-GAAGACAGAGACAGCGGCTT-3'
	Reverse: 5'-ATCTTAAAGGCTGAACTCACTGG-3'
MRC1	Forward: 5'-GGGGAAAGGTTACCCTGGTG-3'
	Reverse: 5'-TCAAGGAAGGGTCGGATCGT-3'
IL-10	Forward: 5'-TGAAAACAAGAGCAAGGCCG-3'
	Reverse: 5'-GCCACCCTGATGTCTCAGTT-3'
CD86	Forward: 5'-AGGCAACAATGAGCAGACCA-3'
	Reverse: 5'-ACTATGGCTTGTTGGGTGGG-3'
IL-12p35	Forward: 5'-CAGAAGGCCAGACAAACTCT-3'
	Reverse: 5'-GCCAGGCAACTCCCATTAGTTA-3'
MSR1	Forward: 5'-CGTTGGACAGGTCGTCTGTA-3'
	Reverse: 5'-CTTGTCCCCCATTGCCGAAT-3'
iNOS	Forward: 5'-ACCTACCAACTGACGGGAGA-3'
	Reverse: 5'-ATGGCCGACCTGATGTTGC-3'
TNF-α	Forward: 5'-CCTCTCTCTAATCAGCCCTCTG-3'
	Reverse: 5'-GAGGACCTGGGAGTAGATGAG-3'
CCL5	Forward: 5'-CCAGCAGTCGTCTTTGTCAC-3'
	Reverse: 5'-CTCTGGGTTGGCACACACTT-3'
CD16	Forward: 5'-GGCTGGGGAAAGGCTGTTTA-3'
	Reverse: 5'-GGGAGATCTTCAGTCCGCAT-3'
TGF-β	Forward: 5'-GGCCAGATCCTGTCCAAGC-3'
	Reverse: 5'-GTGGGTTTCCACCATTAGCAC-3'
CCL2	Forward: 5'-CAGCCAGATGCAATCAATGCC-3'
	Reverse: 5'-TGGAATCCTGAACCCACTTCT-3'

Supplementary Methods

1. Bioinformatics analysis

Kaplan-Meier survival analysis was used to demonstrate the relationship between *CLEC5A* expression and overall survival among glioma patients. We further analyzed genes highly correlated with *CD163* in three GBM datasets using Pearson correlation coefficient analysis. Among these genes, the correlation coefficient of TCGA GBM array dataset and TCGA GBM RNAseq dataset was selected [-0.6–0.6], as was the correlation coefficient of CGGA GBM RNAseq [-0.7–0.7]. Gene Set Enrichment Analysis (GSEA) were performed using the "clusterProfiler" package.

2. Time-dependent ROC curve and survival analysis

Time-dependent ROC curves were used to assess the predictive power of molecular expression to forecast patient outcome in relation to time. We analyzed *CLEC5A* expression using time-dependent ROC curves to analyze of 1- and 2-year survival outcomes. Kaplan-Meier survival analysis indicating the influence of the expression of *CLEC5A* and *CD163* on overall survival time and median value was used as the cutoff to define high and low expression group. Survival analyses were conducted using log-rank (Mantel–Cox) test in GraphPad Prism 10 software.

3. Sample collection, tissue microarray (TMA) and IHC

At the time of tumor banking, glioma tissues were categorized into three regions: tumor center (high cell density, often with necrosis), surrounding tumor (moderate cell density) or tumor border (brain-glioma interface), then paraffin-embedded for IF and IHC analysis. Our TMA contained tissue samples from 55 patients, including 2 non-tumor samples (temporal lobe epilepsy and cortical dysplasia), 1 WHO grade 1 glioma, 12 WHO grade 2 gliomas, 12 WHO grade 3 gliomas and 28 WHO grade 4 gliomas. Each patient sample included tumor center, surrounding tumor and tumor border components. TMA, human tissues and mouse brain tissues were incubated with primary antibody for IHC staining overnight. IHC target proteins were detected using goat anti-rabbit IgG two-step detection kit (PV-9000, ZSGB-Bio, China), then nuclei were counterstained with Mayer's hematoxylin solution (G1080, Solarbio, China). IHC and H&E images were acquired with a VANOX microscope (Olympus, Japan) and IHC intensity score was graded as 0 (negative), 1 (weakly positive, light brown), 2 (moderately positive, brown), or 3

(strongly positive, dark brown). IHC quantity score was graded as 0 (negative), 1 (≤ 25%), 2 (26% - 50%), 3 (51% - 75%), or 4 (> 75%). The total IHC score combines the intensity and quantity scores.

4. Cell culture

Primary human GBM cell lines (N08-74, NU01478, NU01485, NU01805, NU01853, NU02068, NU01594, NU01738 and NU03438) were isolated from human GBM tissue obtained through Emory University and the Nervous System Tumor Bank (NSTB) at Northwestern University, and cultured in Neurobasal medium (21103049, Gibco, USA) supplemented with B-27 supplement (10 ml; 17504044, Gibco, USA), recombinant human bFGF (20 ng/ml; 02634, Stemcell Technologies, Canada), recombinant human EGF (20 ng/ml; 02633, Stemcell Technologies, Canada), heparin (5 ml; 07980, Stemcell Technologies, Canada), GlutaMAX (5 ml; 35050061, Gibco, USA) and Antibiotic-Antimycotic (5 ml; 15240062, Gibco, USA). DF-1 chicken embryonic fibroblasts were generously donated by the laboratory of Oren Becher (Mount Sinai, NY, USA). DF-1 cells were also cultured in DMEM (11965092, Gibco, USA) supplemented with 10% FSB (26140079, Gibco, USA) and Penicillin-Streptomycin (15140122, Gibco, USA). Human monocyte cell line THP-1 was purchased from ATCC (USA), and human peripheral blood monocytes were purchased from Stemcell Technologies (200-0167, Canada). Both were cultured in RPMI 1640 medium (61870036, Gibco, USA) supplemented with 10% FBS and Penicillin-Streptomycin. THP-1 cells were activated using phorbol 12-myristate 13-acetate (PMA, 100 ng/ml, P1585, Millipore Sigma, USA) for 48 hours and polarized towards TAM-IM phenotype using recombinant human IL-4 (25 ng/ml, 200-04, PeproTech, USA) and recombinant human IL-13 (25 ng/ml, 200-13, PeproTech, USA) for another 48 hours. Human peripheral blood monocytes were activated using macrophage colony-stimulating factor (M-CSF, 50 ng/ml, M6518, Millipore Sigma, USA) for 5 days. All cells were incubated in 21% O2 (Normoxic) or 1% O2 (Hypoxic), 5% CO2 at 37℃.

5. Cytokine microarray

THP-1 cells were seeded into the lower chamber of 24-well plate and activated with PMA. After recovery in FBS-free medium, an equivalent number of glioma cells (U87MG) were seeded atop a 3 μm pore membrane in the upper chamber. After 24 hours, co-culture media was collected and cytokines were identified using an antibody microarray kit (QAH-CYT-1, RayBiotech, USA)

according to the manufacturer’s protocol (https://doc.raybiotech.com/pdf/Manual/QAH-CYT-1.pdf).

6. qPCR assay

Total RNA was extracted using TRIzol (15596018, Invitrogen, USA). 5 μg RNA per sample was reverse transcribed to generate a cDNA library using GoScript Reverse Transcription System (A5001, Promega, USA) according to the manufacturer’s instructions. Then, qPCR was performed for 40 cycles to obtain Ct values using GoTaq qPCR Master Mix (A6001, Promega, USA) based on the manufacturer’s protocol. Primer sequences are listed in Table S6.

7. Lentiviral construction and transfection

We constructed shRNA-CLEC5A sequences (sh-CLEC5A-1, 5’-caTTGGCCTAACAAAGACATT-3’; sh-CLEC5A-2, 5’-gtTGTTGGAATGACCTTATTT-3’) and a negative control shRNA sequence (sh-NC, 5’- TTCTCCGAACGTGTCACGT-3’) using the GV493 vector purchased from GeneChem, China. We also generated a CLEC5A overexpression plasmid using GV492 vector with FLAG tag and an empty vector negative control (GeneChem, China). Lentiviral transfection of THP-1 cells was performed according to manufacturer’s protocol, with subsequent selection using 2 μg/ml puromycin (A1113803, Gibco, USA).

8. CT-2A syngeneic orthotopic glioma model

To validate findings in an independent immunocompetent model, a syngeneic CT-2A orthotopic glioma model was established in C57BL/6 mice. Bone marrow transplantation (BMT) was performed using wildtype or *Clec5a*$^{-/-}$ donor mice. Recipient mice were lethally irradiated prior to transplantation and allowed to reconstitute hematopoiesis before tumor implantation. CT-2A glioma cells (2×10^4 cells in 1 μl media) were stereotactically implanted into the brains of BMT recipients. Tumor progression was assessed by MRI and 3D volumetric reconstruction, and survival was monitored longitudinally.
