# Supplementary material for: The perinecrotic niche of glioblastoma drives tumor-associated macrophage polarization and immunosuppression via podoplanin-mediated CLEC5A activation
Source: J Clin Invest. 2026 Jun 2;136(14):e199228. doi: 10.1172/JCI199228 (PMC13367977; doi:10.1172/JCI199228)

Full unedited blot/gel for Fig. 1I

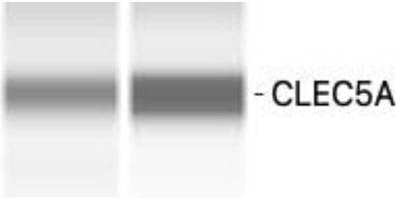

Full unedited blot/gel for Fig. 4C

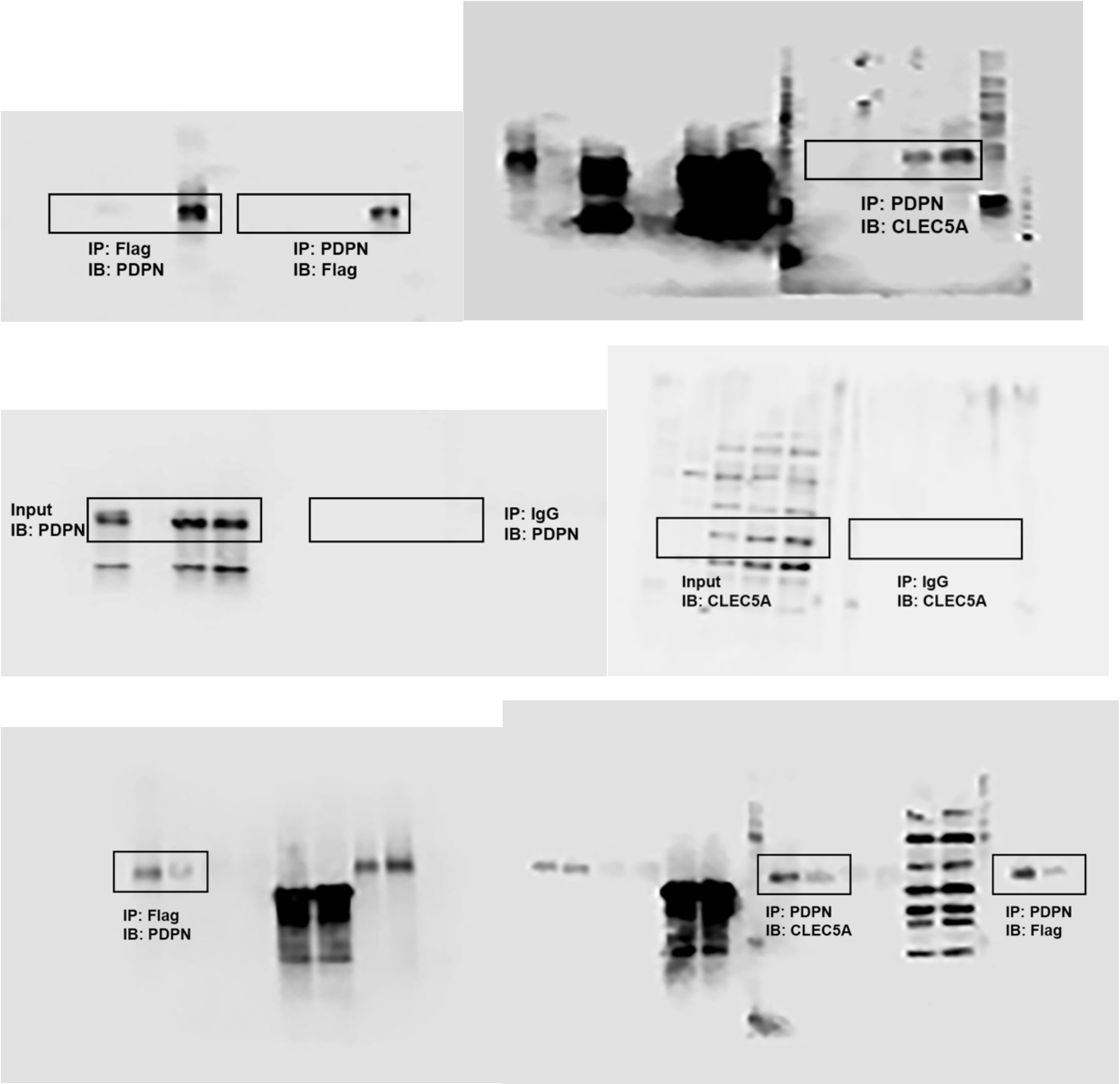

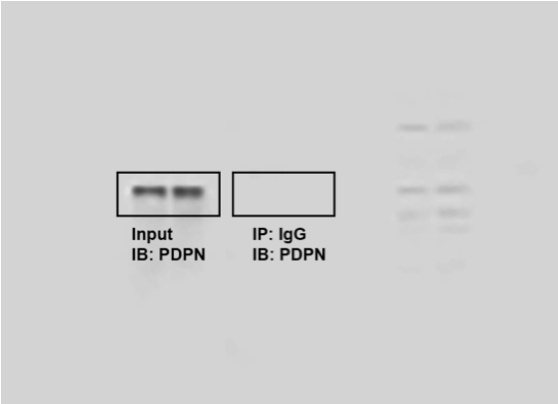

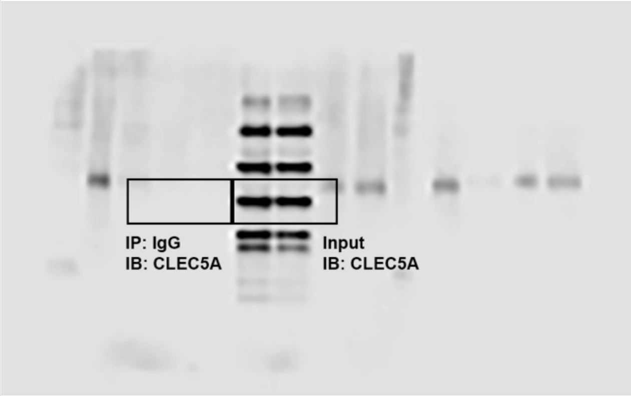

Full unedited blot/gel for Fig. 6C

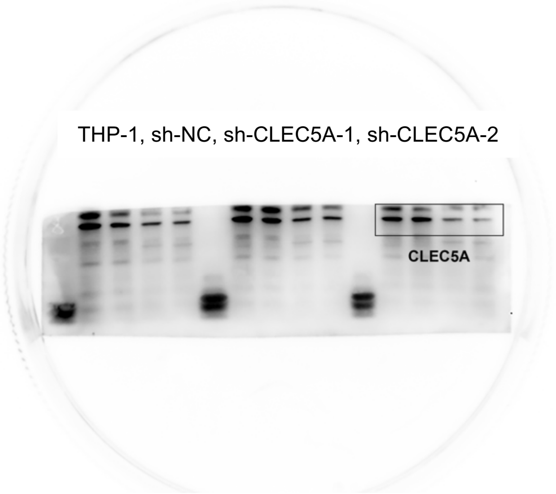

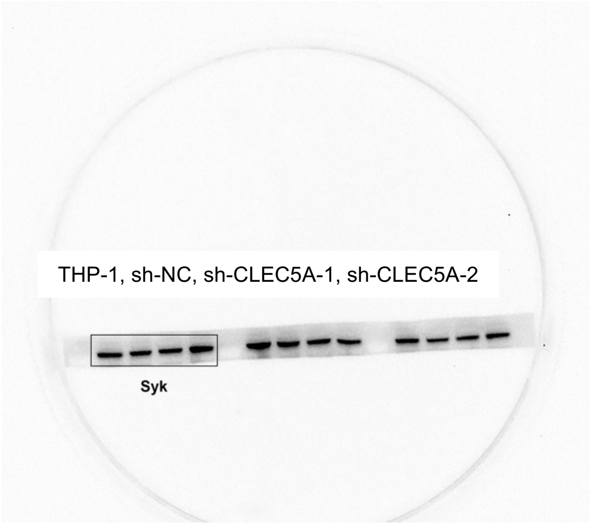

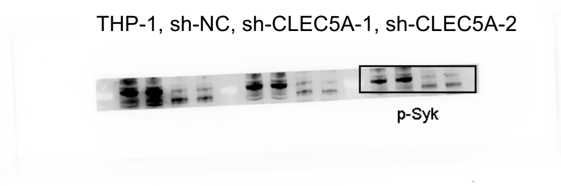

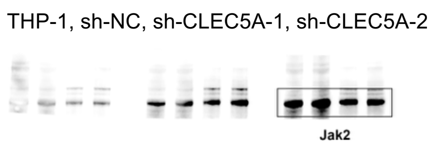

THP-1, sh-NC, sh-CLEC5A-1, sh-CLEC5A-2

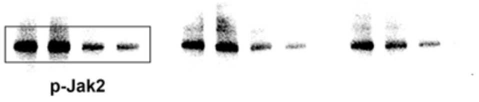

THP-1, sh-NC, sh-CLEC5A-1, sh-CLEC5A-2

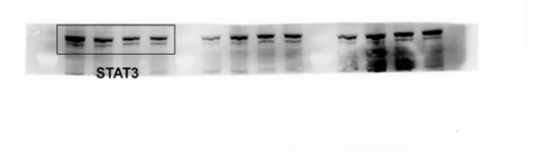

THP-1, sh-NC, sh-CLEC5A-1, sh-CLEC5A-2

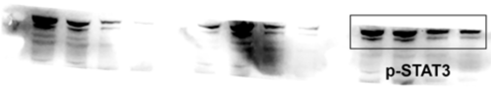

THP-1, sh-NC, sh-CLEC5A-1, sh-CLEC5A-2

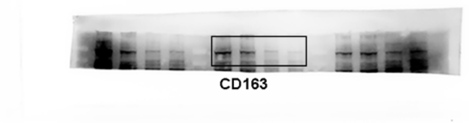

THP-1, sh-NC, sh-CLEC5A-1, sh-CLEC5A-2

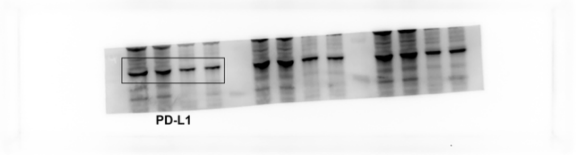

THP-1, sh-NC, sh-CLEC5A-1, sh-CLEC5A-2

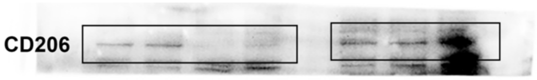

THP-1, EV, OE-CLEC5A

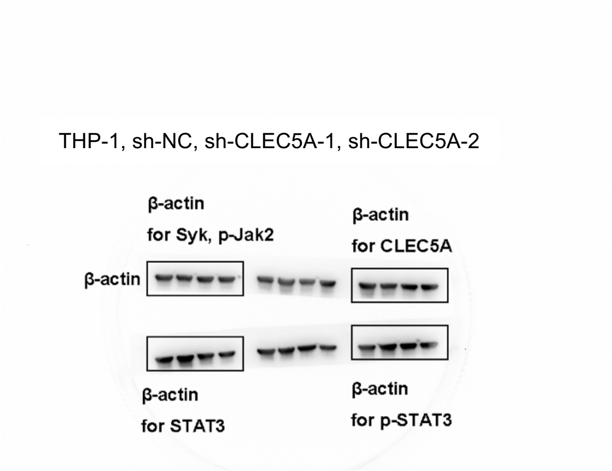

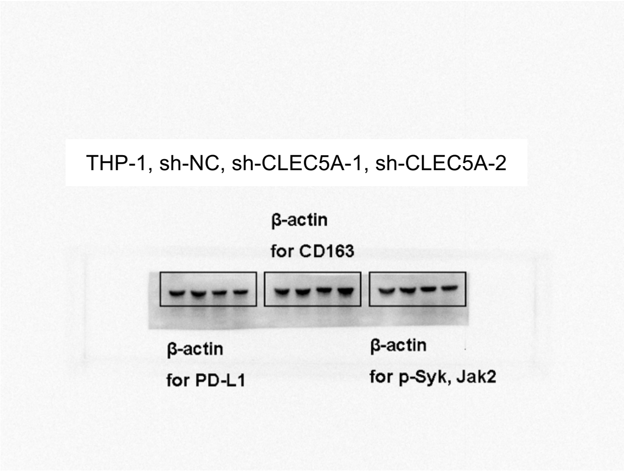

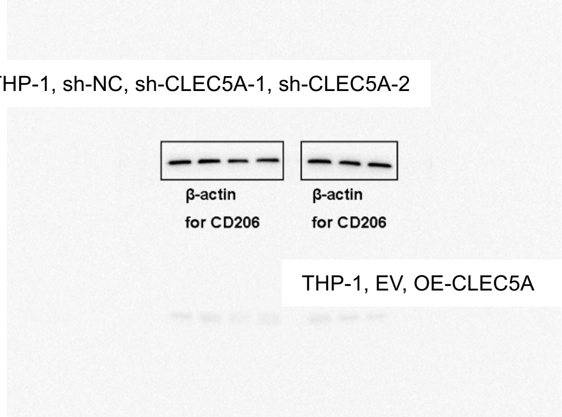

THP-1, EV, OE-CLEC5A

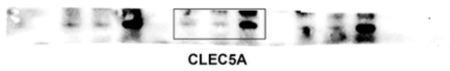

THP-1, EV, OE-CLEC5A

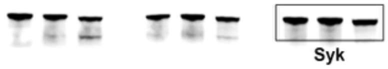

THP-1, EV, OE-CLEC5A

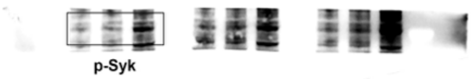

THP-1, EV, OE-CLEC5A

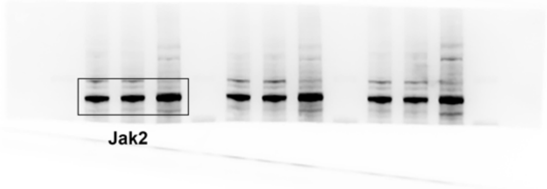

THP-1, EV, OE-CLEC5A

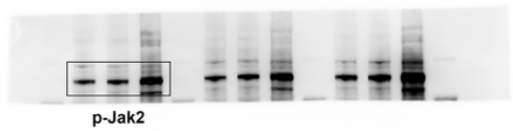

THP-1, EV, OE-CLEC5A

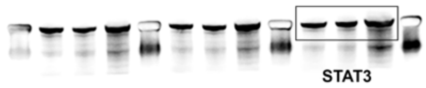

THP-1, EV, OE-CLEC5A

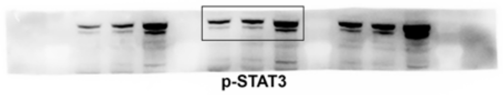

THP-1, EV, OE-CLEC5A

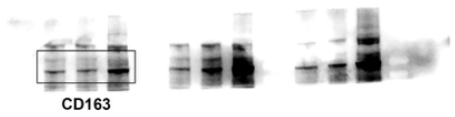

THP-1, EV, OE-CLEC5A

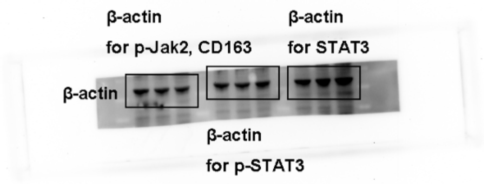

THP-1, EV, OE-CLEC5A

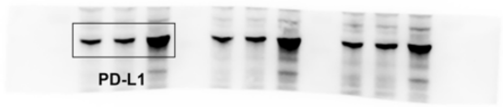

THP-1, EV, OE-CLEC5A

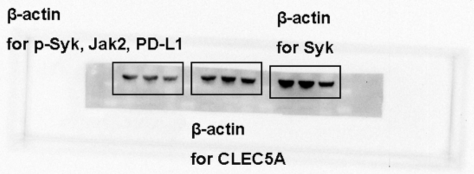

Full unedited blot/gel for Fig. 6F

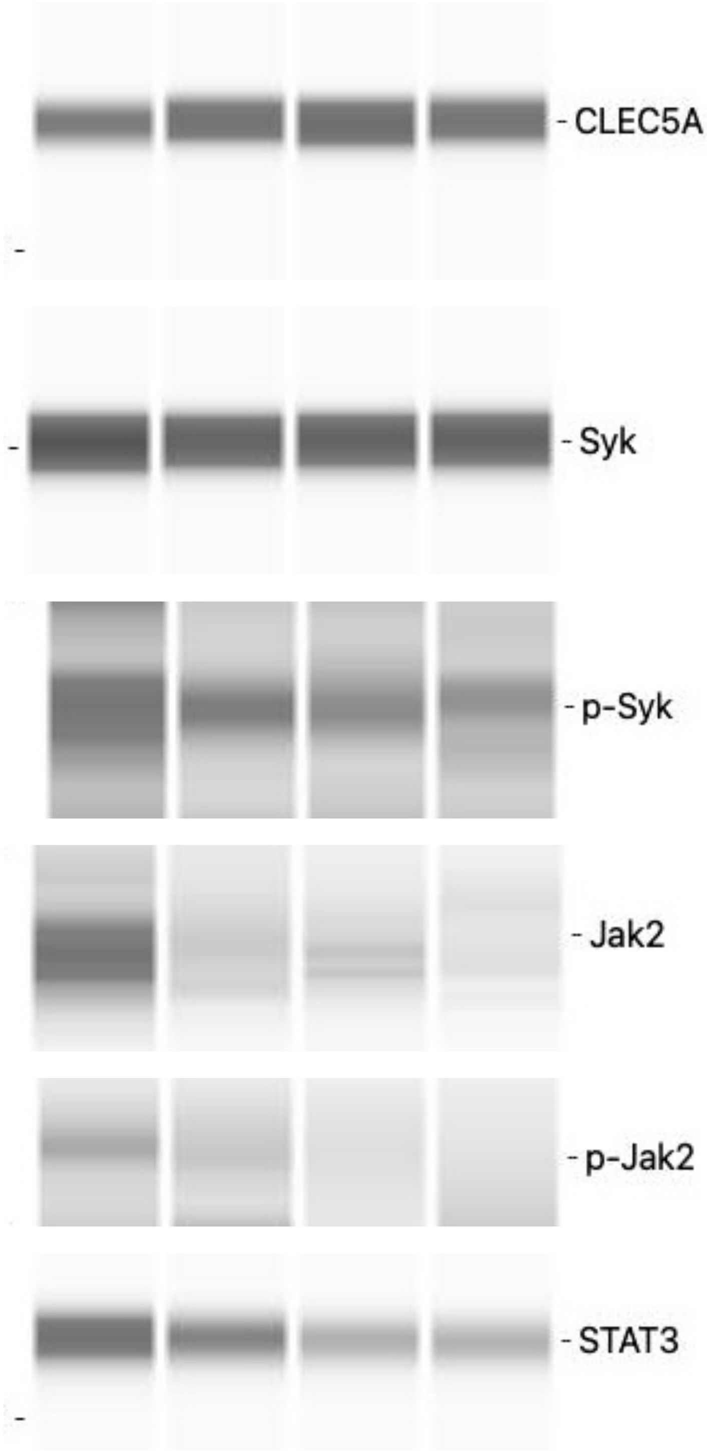

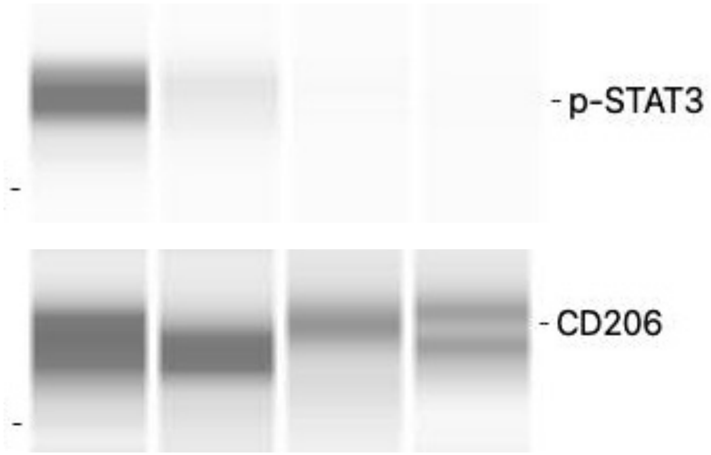

Full unedited blot/gel for Fig. 6G

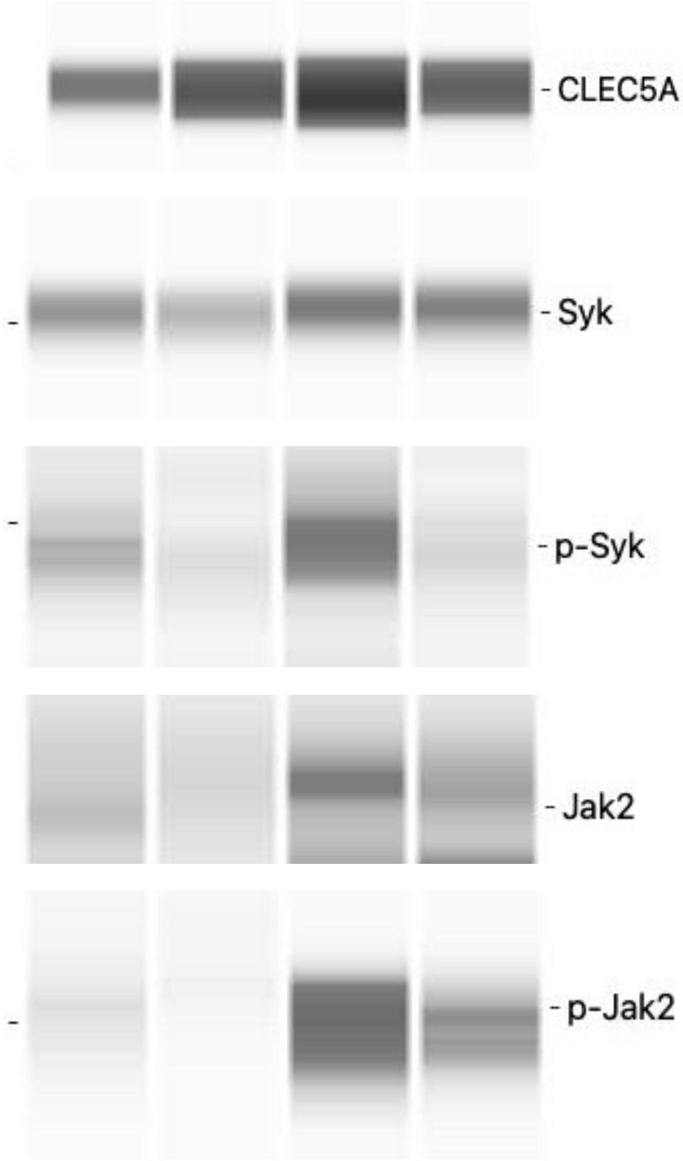

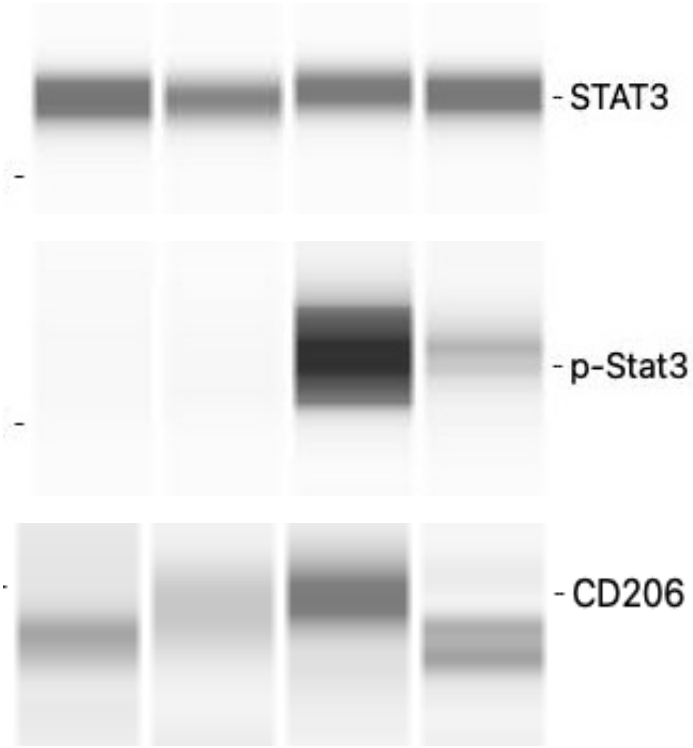

Full unedited blot/gel for SFig. 6H

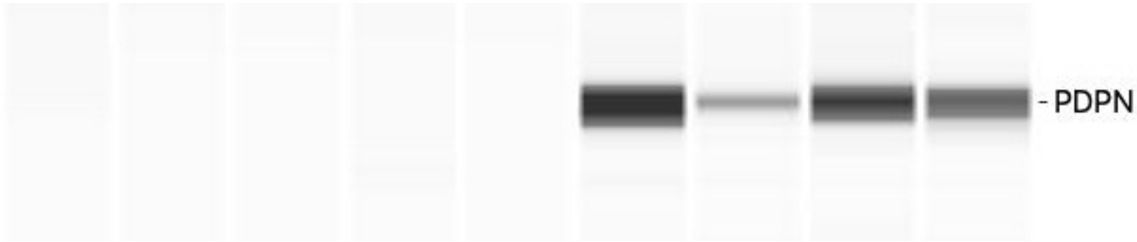

Full unedited blot/gel for SFig. 6I

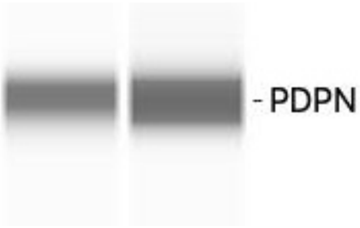

Supplement: Unedited blot and gel images [file jci-136-199228-s009.pdf]
